# Correction: Identifying and overcoming barriers to automated external defibrillator use by GoodSAM volunteer first responders in out-of-hospital cardiac arrest using the Theoretical Domains Framework and Behaviour Change Wheel: a qualitative study

**DOI:** 10.1136/bmjopen-2019-034908corr1

**Published:** 2020-03-19

Smith CM, Griffiths F, Fothergill RT, *et al.* Identifying and overcoming barriers to automated external defibrillator use by GoodSAM volunteer first responders in out-of-hospital cardiac arrest using the Theoretical Domains Framework and Behaviour Change Wheel: a qualitative study. *BMJ Open* 2020;10:e034908. doi: 10.1136/bmjopen-2019-034908.

This article was previously published with an error.

The funding statement has been updated to:

CMS is funded by a National Institute for Health Research (NIHR) Doctoral Research Fellowship (DRF-2017-10-095) for this research project. This publication presents independent research funded by the National Institute for Health Research (NIHR). The views expressed are those of the author(s) and not necessarily those of the NHS, the NIHR or the Department of Health and Social Care.

