# Supplementary material for: Identifying and overcoming barriers to automated external defibrillator use by GoodSAM volunteer first responders in out-of-hospital cardiac arrest using the Theoretical Domains Framework and Behaviour Change Wheel: a qualitative study
Source: BMJ Open. 2020 Mar 10;10(3):e034908. doi: 10.1136/bmjopen-2019-034908 (PMC7066637; doi:10.1136/bmjopen-2019-034908)
Supplement: Supplementary data [file bmjopen-2019-034908supp001.pdf]

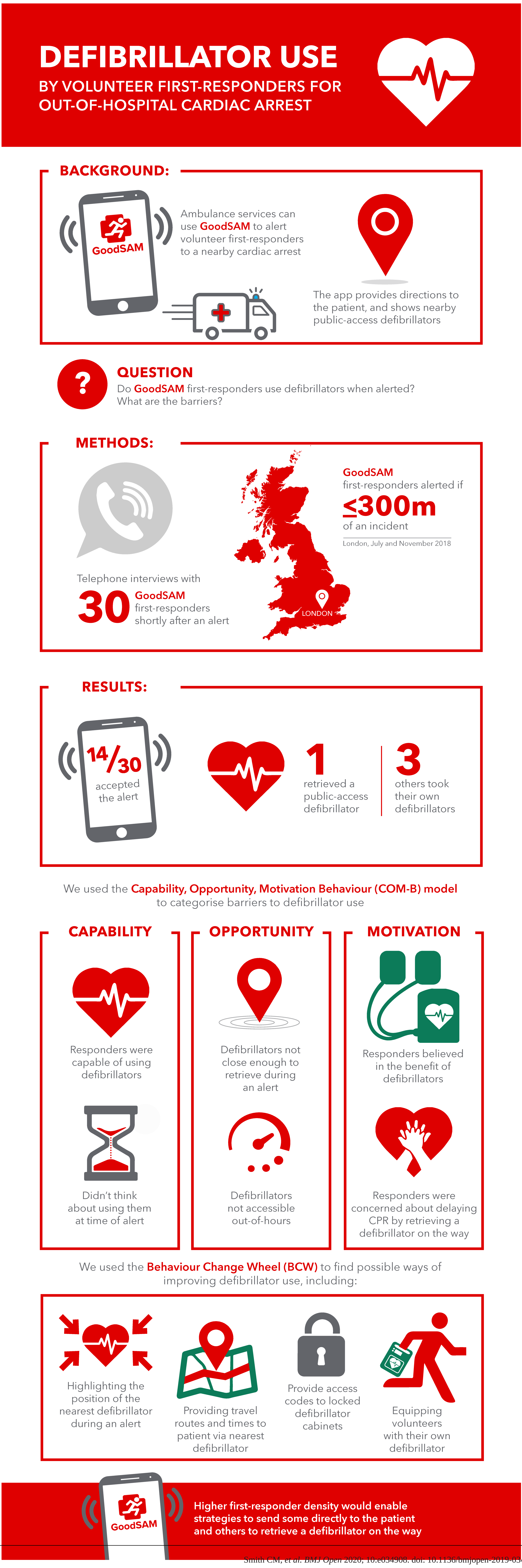
DEFIBRILLATOR USE
BY VOLUNTEER FIRST-RESPONDERS FOR OUT-OF-HOSPITAL CARDIAC ARREST
BACKGROUND:
GoodSAM
Ambulance services can use GoodSAM to alert volunteer first-responders to a nearby cardiac arrest
The app provides directions to the patient, and shows nearby public-access defibrillators
QUESTION
Do GoodSAM first-responders use defibrillators when alerted? What are the barriers?
METHODS:
GoodSAM first-responders alerted if ≤300m of an incident
London, July and November 2018
LONDON
Telephone interviews with 30 GoodSAM first-responders shortly after an alert
RESULTS:
14/30 accepted the alert
1 retrieved a public-access defibrillator
3 others took their own defibrillators
We used the Capability, Opportunity, Motivation Behaviour (COM-B) model to categorise barriers to defibrillator use
CAPABILITY
Responders were capable of using defibrillators
Didn't think about using them at time of alert
OPPORTUNITY
Defibrillators not close enough to retrieve during an alert
Defibrillators not accessible out-of-hours
MOTIVATION
Responders believed in the benefit of defibrillators
Responders were concerned about delaying CPR by retrieving a defibrillator on the way
We used the Behaviour Change Wheel (BCW) to find possible ways of improving defibrillator use, including:
Highlighting the position of the nearest defibrillator during an alert
Providing travel routes and times to patient via nearest defibrillator
Provide access codes to locked defibrillator cabinets
Equipping volunteers with their own defibrillator
GoodSAM
Higher first-responder density would enable strategies to send some directly to the patient and others to retrieve a defibrillator on the way
