# Supplementary material for: Identifying and overcoming barriers to automated external defibrillator use by GoodSAM volunteer first responders in out-of-hospital cardiac arrest using the Theoretical Domains Framework and Behaviour Change Wheel: a qualitative study
Source: BMJ Open. 2020 Mar 10;10(3):e034908. doi: 10.1136/bmjopen-2019-034908 (PMC7066637; doi:10.1136/bmjopen-2019-034908)
Supplement: Author's manuscript [file bmjopen-2019-034908.draft_revisions.pdf]

BMJ Open

BMJ Open is committed to open peer review. As part of this commitment we make the peer review history of every article we publish publicly available.

When an article is published we post the peer reviewers' comments and the authors' responses online. We also post the versions of the paper that were used during peer review. These are the versions that the peer review comments apply to.

The versions of the paper that follow are the versions that were submitted during the peer review process. They are not the versions of record or the final published versions. They should not be cited or distributed as the published version of this manuscript.

BMJ Open is an open access journal and the full, final, typeset and author-corrected version of record of the manuscript is available on our site with no access controls, subscription charges or pay-per-view fees (http://bmjopen.bmj.com).

If you have any questions on BMJ Open's open peer review process please email
info.bmjopen@bmj.com

BMJ Open

Identifying and overcoming barriers to Automated External Defibrillator use by volunteer first-responders responding to nearby out-of-hospital cardiac arrest using the Theoretical Domains Framework and Behaviour Change Wheel.

Journal:	*BMJ Open*
Manuscript ID	bmjopen-2019-034908
Article Type:	Original research
Date Submitted by the Author:	10-Oct-2019

Complete List of Authors:	Smith, Christopher Matthew; University of Warwick, Warwick Clinical Trials Unit<br>Griffiths, Frances; University of Warwick, Warwick Medical School<br>Wilson, Mark; Imperial College London, Biomedical Research Centre<br>Fothergill, Rachael; London Ambulance Service NHS Trust, Clinical Audit and Research Unit<br>Vlaev, Ivo; Warwick Business School,<br>Perkins, Gavin; University of Warwick, Warwick Clinical Trials Unit

Keywords:	QUALITATIVE RESEARCH, PUBLIC HEALTH, ACCIDENT & EMERGENCY MEDICINE

SCHOLARONE™
Manuscripts

I, the Submitting Author has the right to grant and does grant on behalf of all authors of the Work (as defined in the below author licence), an exclusive licence and/or a non-exclusive licence for contributions from authors who are: i) UK Crown employees; ii) where BMJ has agreed a CC-BY licence shall apply, and/or iii) in accordance with the terms applicable for US Federal Government officers or employees acting as part of their official duties; on a worldwide, perpetual, irrevocable, royalty-free basis to BMJ Publishing Group Ltd ("BMJ") its licensees and where the relevant Journal is co-owned by BMJ to the co-owners of the Journal, to publish the Work in this journal and any other BMJ products and to exploit all rights, as set out in our licence.

The Submitting Author accepts and understands that any supply made under these terms is made by BMJ to the Submitting Author unless you are acting as an employee on behalf of your employer or a postgraduate student of an affiliated institution which is paying any applicable article publishing charge ("APC") for Open Access articles. Where the Submitting Author wishes to make the Work available on an Open Access basis (and intends to pay the relevant APC), the terms of reuse of such Open Access shall be governed by a Creative Commons licence – details of these licences and which Creative Commons licence will apply to this Work are set out in our licence referred to above.

Other than as permitted in any relevant BMJ Author's Self Archiving Policies, I confirm this Work has not been accepted for publication elsewhere, is not being considered for publication elsewhere and does not duplicate material already published. I confirm all authors consent to publication of this Work and authorise the granting of this licence.

For peer review only

Identifying and overcoming barriers to Automated External Defibrillator use by volunteer first-responders responding to nearby out-of-hospital cardiac arrest using the Theoretical Domains Framework and Behaviour Change Wheel.

Christopher M Smith [1] (corresponding author: c.smith.20@warwick.ac.uk)

Frances Griffiths [2]

Mark Wilson [3]

Rachael Fothergill [4]

Ivo Vlaev [5]

Gavin D Perkins [1]

[1] Warwick Clinical Trials Unit, University of Warwick, Coventry, CV4 7AL, UK

[2] Division of Health Sciences, Warwick Medical School, University of Warwick, Coventry, CV4 7AL, UK

[3] Imperial College Biomedical Research Centre, St Mary's Hospital, London, W2 1NY, UK

[4] Clinical Audit and Research Unit, London Ambulance Service NHS Trust, London, SE1 0BW

[5] Warwick Business School, University of Warwick, Coventry, CV4 7AL, UK

Keywords: Out-Of-Hospital Cardiac Arrest; defibrillators; cardiopulmonary resuscitation; qualitative research

For peer review only

For peer review only - http://bmjopen.bmj.com/site/about/guidelines.xhtml

Abstract

Objectives: GoodSAM is a mobile-phone app that integrates with UK ambulance services. During a 999 call, if a call-handler diagnoses cardiac arrest, nearby volunteer first-responders registered with the app receive an alert. First-responders can give CPR and/or defibrillation using a public-access Automated External Defibrillator (AED). We used the Theoretical Domains Framework to identify and classify barriers to AED use by GoodSAM first-responders, and subsequently the Behaviour Change Wheel to develop potential interventions to improve AED use.

Design: Semi-structured telephone interviews, recorded using Skype (audio) seeking to understand Capability, Opportunity and Motivation of first-responders.

Setting: London, UK.

Participants: GoodSAM first-responders who received an alert in the previous seven days

Results: We conducted 30 telephone interviews in two batches in July and October 2018. A public-access AED was taken to scene once, one had already been attached on scene on another occasion, and three participants took their own AEDs when responding to an alert. Most first-responders felt capable and motivated to use public-access AEDs, but concerned about delaying CPR if they went to retrieve one, and frustrated when they arrived after the ambulance service. They perceived lack of opportunities to use public-access AEDs related to unavailability and inaccessibility, particularly out-of-hours. We subsequently developed thirteen potential interventions to increase AED use, for future testing.

For peer review only - http://bmjopen.bmj.com/site/about/guidelines.xhtml

Conclusions: GoodSAM first-responders used AEDs occasionally, despite a capability and motivation to do so. Those operating volunteer first-responder systems should consider our proposed interventions to improve AED use. Highlighting AED location and providing route and time estimates to the patient via the nearest AED would assist single responders to decide about retrieving an AED. As AED collection may extend the time taken to reach the patient, where there is sufficient density of potential responders, first-responder systems could send one responder to initiate CPR and another to collect an AED.

Abstract word count: 300

For peer review only

Strengths and limitations of this study

- This interview study is the first time behaviours affecting Automated External Defibrillator (AED) use of volunteer first-responders activated to out-of-hospital cardiac arrest have been explored using the Theoretical Domains Framework and Behaviour Change Wheel
- We have developed a list of potential interventions to increase AED use that can be tested by *any* similar first-responder system, of which there are a number worldwide
- Actual AED use in this study was infrequent: interview responses focus on intended actions and may not be representative of participants' behaviours in future time-critical emergency situations

review only

For peer review only - http://bmjopen.bmj.com/site/about/guidelines.xhtml

Introduction

Fewer than one in ten patients who suffer an out-of-hospital cardiac arrest (OHCA) survive to hospital discharge in England (1). Public Access Defibrillation (PAD) – bystander use of public-access Automated External Defibrillators (AEDs) – is associated with doubled survival to hospital discharge with good neurological function (2). However, PAD is used fewer than 5% of OHCA (1,3).

Volunteer first-responder systems

Mobile app-based volunteer first-responder systems are one approach to overcome limited PAD use (4). A number of countries have app-based or text-message systems to alert bystanders to a nearby OHCA, including Netherlands (5), Sweden (6) and USA (7). 'GoodSAM' (https://www.goodsamapp.org/) is the system that operates in the UK (8). Patient outcome data are limited (9). The only randomised controlled trial, comparing a text-message alert system supplementing ambulance service care vs ambulance service care alone, reported significantly higher bystander CPR rates (primary outcome: 61.6% vs 47.8%, p<0.001) but no significant difference in the secondary outcome of 30-day survival (11.2% vs 8.6%, p=0.28). However, there was no mention of PAD in this system (6). In an observational study in the Netherlands (2012-2014), OHCA patients attended by text-message-alerted first-responders were 2.8 times more likely to survive than those for whom alerted first-responders did not attend. In this system, first-responders attached an AED in 26.8% cases (5).

For peer review only - http://bmjopen.bmj.com/site/about/guidelines.xhtml

Barriers and facilitators to PAD

A systematic review considering barriers and facilitators to PAD reported that public-access AEDs are located close to few OHCA, and their accessibility is reduced out-of-hours. Knowledge, awareness and willingness to use public-access AED vary, but are increased by prior CPR/AED training (3). In volunteer first-responder systems, not reaching the scene after accepting an alert (5,7,10), not being the first to arrive and perform CPR (7,11) or attach an AED (10) have all been reported.

Psychological distress does occur in bystanders, but it is rare that people require professional support (12,13). In a Dutch text-message alert system, 13% responders reported severe short-term impact on their psychological heath. However, by four to six weeks, all reported either no (81%) or mild (19%) stress only (14).

There is limited knowledge about the effect of first-responder systems and how to most effectively deploy AEDs within them, and recognition that we need high-quality research to investigate this (4). Currently, the Scandinavian AED and Mobile Bystander Activation (SAMBA) trial (15) is randomising first-responders using the 'Heartrunner' volunteer first-responder app in Denmark and Sweden to either: all responders travel direct to the patient or; some responders retrieve the nearest AED first. The primary outcome is the proportion of patients who have an AED attached before the ambulance service arrives.

For peer review only - http://bmjopen.bmj.com/site/about/guidelines.xhtml

Theoretical frameworks to develop interventions for increased PAD use

There is no work using a theoretically-informed behavioural framework to address low AED use in volunteer first-responder systems. The Theoretical Domains Framework (TDF) can be used to identify specific barriers or facilitators to a particular outcome (16,17). The 14 domains of the revised version of the TDF (18) can be further grouped and integrated with the COM-B (Capability, Opportunity, Motivation) behavioural framework. COM-B characterises three targets for behavioural change in order to inform the design of healthcare interventions and can, in turn, be linked to the Behaviour Change Wheel (BCW) (19). The BCW is used to develop interventions and the means of implementing them.

Study aims

Using the BCW, we aimed to take data from an interview study with GoodSAM first-responders (focussing primarily on whether or not to retrieve an AED before going to the patient) and existing evidence from the literature, to develop a theoretically-informed list of interventions to increase public-access AED use by GoodSAM first-responders.

For peer review only - http://bmjopen.bmj.com/site/about/guidelines.xhtml

Methods

Description of GoodSAM system

GoodSAM is an app-based system that integrates with several local ambulance services to alert trained volunteers to an OHCA (8). A call-handler, using a Computer Aided Dispatch (CAD) system during a 999 call, allocates a code identifying the clinical problem. If this code indicates that a patient is suffering a current or imminent cardiac arrest, the system automatically activates GoodSAM. First-responders can accept or decline this alert, and receive an incident address and route. The app also displays nearby public-access AEDs. In July 2018, GoodSAM responders accepted 19% of alerts (information from GoodSAM).

Study Setting

London, UK. GoodSAM integrated with the London Ambulance Service CAD system in October 2015. Following an OHCA diagnosis during the 999 call, GoodSAM first-responders within 300m of the incident receive an alert siren on their mobile device.

Study design

Telephone semi-structured interviews performed with GoodSAM first-responders in London, analysed using the TDF. We developed topic guides based on COM-B. The focus in first-responder interviews was on decisions to accept (or decline) their most recent alert, and to use a public-access AED.

For peer review only - http://bmjopen.bmj.com/site/about/guidelines.xhtml

Participant selection and recruitment

We approached first-responders consecutively in two blocks in July and October 2018, shortly after they received an alert in London. We made an a priori decision to conduct 30 interviews before assessing for data saturation – defined here as no new information emerging from interviews 28-30 (20). We felt this was pragmatic and consistent with other studies using qualitative interviews (21).

Interview procedure

We approached potential participants via e-mail, with a participant information sheet and consent statements attached. Interested participants replied to CMS, who subsequently arranged an interview. We sent no further e-mails to non-responders.

CMS conducted interviews with GoodSAM first-responders using Microsoft Skype (audio only) to the telephone number provided, and recorded directly onto computer using QuickTime player. CMS introduced himself as a clinician but explained his current role as a University researcher undertaking a PhD. Interviewer and participants did not previously know one another. Participants could ask questions before recording started. Recording began and participants gave verbal agreement to consent statements, which the interviewer read aloud. Interviews lasted approximately 15 minutes, and recording continued until the call ended. All interviews took place at least 24 hours after the initial invitation, and within seven days of the GoodSAM alert.

For peer review only - http://bmjopen.bmj.com/site/about/guidelines.xhtml

We kept audio files and transcriptions in separate encrypted computer folders. No participant-identifying information was associated or stored with audio files or transcripts.

Interview data analysis

CMS transcribed and coded interviews using NVivo (QSR International). CMS and FG subjected the first three interviews to detailed line-by-line coding without reference to the TDF: they judged that the codes and emerging themes sufficiently matched with TDF domains for us to comprehensively code information to these TDF domains. CMS subsequently coded all interviews using the TDF, with periodic checking undertaken by FG and iterative update and review of coding as required. We remained alert to data that did not fit into a TDF domain. We matched coded material from TDF domains to components of COM-B, with which the TDF integrates (19). We subsequently analysed the data and synthesised a narrative about the first-responders' experiences relating to their capability (subdivided into physical and psychological), opportunity (social and physical) and motivation (automatic and reflective) to use an AED as part of their response.

Developing interventions

We developed potential interventions to improve AED use by GoodSAM first-responders, using a step-by-step approach described in the BCW (19). We identified relevant intervention functions for behavioural determinants that would have to change to increase AED use. We further assessed these intervention functions (from a list of

For peer review only - http://bmjopen.bmj.com/site/about/guidelines.xhtml

nine in the BCW) using APEASE (Affordability, Practicability, Effectiveness/cost-effectiveness, Safety, Efficacy) criteria and identified policy categories, behavioural change techniques and modes of delivery. We selected behavioural change techniques described in the Behaviour Change Techniques Taxonomy (BCTT) version 1 (22): these associate with intervention functions in the BCW (19).

Patient and Public Involvement

There were two lay representatives on the project's advisory committee. They ontributed to the topic guide, participant information sheets, consent forms and protocol.

Ethics

The study received ethical approval from the Biomedical Sciences Research Ethics Committee at the University of Warwick on 16/03/2018 (ref: REGO-2018-2164).

Reporting

We report this study according to Standards for Reporting Qualitative Research (23) (**supplementary file**)

For peer review only

Results

1. Interview Study

We conducted 30 telephone interviews, from 248 e-mail invitations. Twenty-one interviews took place in July 2018. After reviewing the topic guide we conducted the remaining interviews in October 2018. A higher proportion of our first twenty-one participants had accepted their latest alert (12/21, 57%) compared to the London average, so for these last nine interviews we invited only those who had declined their latest alert. We determined that we had achieved data saturation after 30 interviews.

Considering their most recent alert, fourteen (47%) responders accepted. Six reached the patient's side; three before the ambulance service. Four of eleven patients, where known, were in cardiac arrest. Most participants had also received previous alerts so we also discussed issues relating to these alerts, starting with the most recent.

We present a summary of barriers and facilitators to AED use in **Figures 1-3.**

Physical capability

No participant said they felt unable to provide CPR or use an AED, and all who commented believed previous CPR/AED training facilitated CPR and AED use when responding. Eight participants reported that real-life experience of OHCA would help them effectively use an AED in future responses.

For peer review only - http://bmjopen.bmj.com/site/about/guidelines.xhtml

Psychological capability

Participants were knowledgeable about public-access AEDs, likely locations and that the app displayed them:

> "I'm aware that tube stations, Pret, the usual kind of suspects will have them, so there's quite a few within close proximity, not on my route, but if I needed help I could direct someone, go to just up the street, go to Boots" (#10)

Three participants reported knowledge of AEDs on the app that were unavailable out-of-hours (#12, #14, #24). Six participants knew about an AED's location only after browsing the app (i.e. when *not* receiving an alert). Four participants said they were less knowledgeable about AED locations outside their home area:

> "Most places I wouldn't [know where an AED was] unless I looked on the GoodSAM app and it was on the map." (#20)

Nine reported not remembering whether the app displayed a nearby AED at the time of the alert; seven others reported that they remembered seeing an AED when alerted.

Social Opportunity

One participant (who had declined every alert that he had received) did not feel pressured to act by other bystanders, as the patient was somewhere else:

For peer review only - http://bmjopen.bmj.com/site/about/guidelines.xhtml

> "It's easier to be influenced I think by a lack of confidence if it's not a real human being in front of you." (#21)

Physical Opportunity

Some app-specific issues affected participants' ability to accept an alert. Three reported that it was slow to load (#16; #19; #25) or provide incident location (#25). Seven participants reported that GoodSAM incorrectly recorded their location at the time of an alert.

No participant reported problems accessing the patient when they were first on-scene. However, eleven participants arrived after the ambulance service on their most recent alert. Three participants (#14; #17; #25) reported this on earlier alerts as well.

Nine participants reported on their interaction with ambulance service personnel. Five participants (#5; #16; #22; #23; #24) reported helping on scene:

> "Once he realised the level of training that I have he was very excited about the fact that I was there." (#22)

However, three participants (#15; #17; #23) reported occasions when the ambulance service did not accept assistance. Two participants (#14; #23) additionally reported that ambulance personnel were cautious about letting them help, particularly without identification.

For peer review only - http://bmjopen.bmj.com/site/about/guidelines.xhtml

Five participants felt that a standard means of identifying oneself as a first-responder would be useful. Seven participants were concerned about how they would find and negotiate with an AED's custodian for its removal to a different area:

> "There's a whole rigmarole, can I have a defibrillator, I'm a GoodSAM volunteer, are these people going to know?" (#17)

Participant 21 expressed a contrasting view:

> "I kind of imagine if ever an organisation or a building or whatever has an AED and you ran in, you said somebody's having a cardiac arrest can I borrow your AED, I don't imagine that many people would say no." (#21)

A public-access AED was taken to a patient once when considering participants' most recent alerts, by participant #3, who obtained it from their workplace. Participant #30 found an AED already attached to the patient on-scene. Three participants (#6; #13; #16) took their own AEDs to the scene during their most recent alert. Eight participants reported that there were no public-access AEDs close enough to retrieve when they accepted an alert.

Two other participants (#26; #30) believed that finding an AED's exact location would be difficult. Participant 26 was unsure how one would retrieve an AED that was kept in a code-locked cabinet (which many are), and whether or not this code would be

For peer review only - http://bmjopen.bmj.com/site/about/guidelines.xhtml

available through the app. Contrastingly, two participants (#5; #13) believed that AEDs would not be too difficult to find:

> "I think most of them are fairly easy accessible, I think they generally have to be in well-located places and easy to find." (#13)

Four participants commented on there being fewer public-access AEDs available out-of-hours:

> "They're in GP surgeries or local shops and both of the times I've responded they've been out-of-hours." (#12)

Automatic motivation

Seven participants reported anxiety about on-scene management, including dealing with patients who have not suffered a cardiac arrest:

> "I suppose if there was like mental health issues, or drugs and alcohol involved, I would be a bit apprehensive like getting involved in that" (#6)

Three participants (#8; #14; #23) expressed concern about what had happened to a patient when they did not accept an alert:

> "I did have that real nagging feeling afterwards about 'oh my god, you know, that person could've died because I didn't go" (#14)

For peer review only - http://bmjopen.bmj.com/site/about/guidelines.xhtml

For peer review only

Two participants (#6; #28) said that they would welcome incident feedback, or a "debrief" from the ambulance service (#9).

Reflective motivation

Nine participants said that GoodSAM was an important initiative. Three (#6; #9, #16) expressed the belief that it has already saved lives.

Several factors affected or would affect the intention to accept an alert. Eleven participants cited distance to the incident. Six participants reported a belief that ambulance personnel would arrive before them, reinforced by previous experience:

> "I find that very frustrating, because you drop what you're doing, go and assist someone and then by the time you get there there's already enough people so you just kind of not go." (#14)

One participant (#30) was informed by the app about other GoodSAM first-responders accepting or declining the same alert, but did not believe this had affected the decision to respond. Twenty participants stated a preference for going directly to the patient to assess the situation and provide CPR, rather than retrieving an AED first:

> "[An AED] obviously makes a massive difference to early survival, but I think I would deem somebody doing good-quality chest compressions as

For peer review only - http://bmjopen.bmj.com/site/about/guidelines.xhtml

> a higher priority than taking an extra five to ten minutes to find the local machine.” (#26)

Negotiating access to an AED from its custodian might also affect the motivation to retrieve one:

> “I wouldn’t waste too much time… If I couldn’t get it immediately within 5 to 10 seconds I would be on my way without it.” (#19)

Seven participants commented on the importance of early AED use to survival, and three (#10; #18; #20) said they would look for an AED first if possible:

> “That’s the best way to save their life really. It would be worth the extra minute to get the defibrillator first.” (#18)

Three participants (#13; #17; #21) talked about the uncertainty about whether or not to retrieve an AED first:

> “I appreciate obviously getting an early shock as quick as possible is preferable for the patient but equally I would feel bad if there was someone not doing any compressions and I had to spend five minutes trying to find a defib.” (#13)

For peer review only

For peer review only - http://bmjopen.bmj.com/site/about/guidelines.xhtml

Thirteen participants reported that an AED's proximity to the patient or the route taken to the scene would affect their intention to retrieve it. Five participants said they had prepared by finding AED locations on the GoodSAM app before receiving an alert.

Seventeen participants explicitly expressed confidence in AED use. Reasons given were previous training (#15; #18), previous experience (#3; #6; #10; #11) and perceived ease of use (#11; #15; #18). On-scene issues could affect AED use:

> "Yeah I would be able to do that [use an AED], I think 90%. My hesitancy would be about dealing with the patient and the people around them. To suddenly say right we've got to get this top off and I need to connect these things straight away… So, let's say 80% confident." (#9)

Three participants (#22; #24; #28) remarked that their sense of professional duty affected their motivation to respond to a GoodSAM alert:

> "It's somewhat a large portion of what I do and who I am, so there's not many situations where I wouldn't want to respond to." (#24)

However, three responders (#5, #22, #23 – all healthcare professionals) stated concern about intervening outside their usual environment:

> "It was a little bit more nerve-wracking because you walk in and you're like I want to do this, this and this but actually I can only do CPR." (#5)

For peer review only - http://bmjopen.bmj.com/site/about/guidelines.xhtml

2. Developing interventions to improve AED use by GoodSAM first-responders

We identified ten behavioural determinants that needed to change to increase AED use, thirteen potential interventions to achieve this, the behavioural change techniques required to implement them and how to deliver them **(Table 1)**. We drew upon our knowledge of the existing literature as well as findings from the interview study to develop the intervention list.

Potential interventions were: delivering digital CPR/AED training; providing reminders about nearby AED locations; highlighting the location of the nearest AED at the time of an alert; providing access codes to AEDs in locked cabinets; providing standardised information to show AED custodians when negotiating for an AED's release; streamlining incident location and travel route information provided at the time of an alert; equipping GoodSAM first-responders with their own AEDs; providing reminders about the appropriate use of AEDs; providing distance and time estimates, if travelling to the patient via the nearest AED, at the time of the alert; sending some responders directly to the patient and some via a nearby AED; providing time-to-patient (for first-responder) and ambulance response-time estimates at the time of the alert; delivering motivational messages related to AED use; offering voluntary debrief after an alert.

We have also produced an infographic (**supplementary file**) explaining the study and its main findings.

For peer review only - http://bmjopen.bmj.com/site/about/guidelines.xhtml

For peer review only

Discussion

Principal Findings

We identified thirteen interventions that might increase AED use. Two are primarily concerned with increasing capability, five with increasing opportunity and six with increasing motivation. Some of these interventions also concern means of increasing acceptance of alerts. The two are inexorably linked: there can be no AED use if the first-responder has declined the alert.

Our findings suggest a potential to improve AED use during a GoodSAM response, and these may be relevant to other volunteer first-responder systems. In the interview study, one GoodSAM first-responder retrieved a public-access AED after an alert. In almost all cases participants reported a capability and motivation to provide CPR and use an AED. Whilst knowledgeable about PAD, they saw location and accessibility (particularly out-of-hours) of public-access AEDs as barriers to use. There was concern about the time required to retrieve an AED and not knowing if bystanders were performing CPR during this time. Participants also used the app to familiarise themselves with location and access hours for public-access AEDs. Many reached the patient after the ambulance service and this could affect motivation to respond to future alerts.

For peer review only

For peer review only - http://bmjopen.bmj.com/site/about/guidelines.xhtml

Strengths and Weaknesses of the Study

To our knowledge, this is the first study reporting a theoretically-informed analysis of behaviours affecting AED use in a volunteer first-responder system. We have additionally developed potential interventions for testing that all those who operate similar first-responder systems worldwide can consider. As a public-access AED was retrieved on only one occasion, we lack information about the experience of deciding to retrieve and then use a public-access AED, but this provides justification to develop and test interventions to increase AED use.

Our interview sample may represent GoodSAM first-responders who are more confident and motivated to respond, and so more likely to consent to interview. However, our recruitment method left us unable to explore the characteristics of those who did not reply to the invitation e-mail. With a sensitive topic, participants may have been more likely to tell the interviewer what he wanted to hear (24). CMS is a clinician and doctoral research student but did not offer clinical advice or feedback. However, this could affect how participants responded (25), or how he interpreted information imparted at interview (26).

Comparison with the literature

We have presented a number of novel findings. In addition, this study support previous findings about the lack of appropriately placed AEDs, particularly out-of-hours (3). Our participants reported about not arriving first on scene and/or not intervening in an

For peer review only - http://bmjopen.bmj.com/site/about/guidelines.xhtml

OHCA, in similarity with reports from other volunteer first-responder systems (7,10,11). Volunteer first-responders in a Dutch system also reported anxiety and stress (14).

Implications for clinicians and policymakers

This research suggests that even capable and motivated volunteer first-responders rarely use public-access AEDs. We can reduce physical restraints to AED retrieval by means such as highlighting the location of AEDs, providing access codes to locked cabinets and displaying route distance and time estimates to the nearest AED and to the patient. However, a key concern about diverting to the nearest AED may always be that this increases the length of time that an OHCA patient does not receive CPR. The decision about the appropriateness of such a strategy remains that of the first-responder at the time of the alert.

A key goal when establishing GoodSAM in London was the provision of early CPR, rather than a specific focus on diverting GoodSAM first-responders to retrieve an AED. Patients will not always be in cardiac arrest (four of eleven were in this study, where known) and responders will not always arrive first. This would represent a large number of occasions when an AED was retrieved for no benefit. There was a non-intentional delay of 1-4 minutes in activating GoodSAM in London after a call-handler established a potential OHCA, which became apparent and was rectified only after completion of this study. Reducing the time between the emergency call receipt and first-responder activation, and other strategies such as providing information of expected arrival times of the statutory ambulance service, may minimise the chances of a responder's assistance not being required once they reach the scene.

For peer review only - http://bmjopen.bmj.com/site/about/guidelines.xhtml

Early CPR is vital, so any intervention to improve AED use must not unduly delay CPR. This is possible where AEDs are easily available or if more than one person responds, with one going direct to the patient and another collecting and AED. This is likely to require more volunteers registered with the platform. AEDs can also be dispatched to the scene using taxi drivers (27) or unmanned aerial vehicles (28,29).

Future research

Delivering many of the proposed interventions requires a sufficient number and density of first-responders. We will investigate with stakeholder groups means of doing this, including improved recruitment strategies and if the need for prior CPR certification is an absolute for registration with the app. Not all volunteer first-responder systems require this. Indeed, it varies between different ambulance services using GoodSAM – in New Zealand, prior CPR certification is not required to register. Research priority exercises with GoodSAM, local ambulance services and service users will help us prioritise those interventions that could be tested before an increase in responder numbers, such as highlighting AED location and providing access codes to locked cabinets. These allow existing GoodSAM first-responders to make an informed decision about whether or not to retrieve an AED.

Conclusion

A first-responder used a public-access AED on one occasion in this study. An AED was already attached to the patient on another occasion, and three others took their

For peer review only - http://bmjopen.bmj.com/site/about/guidelines.xhtml

own AEDs to the scene. Despite a capability and motivation to use an AED, interview participants perceived a lack of opportunity to do so. Most believed going to the patient first to assess CPR provision was more beneficial to the patient than diverting to retrieve an AED first. Stakeholders may share this view, but there may still be the potential to improve AED use without compromising CPR provision, particularly if there are multiple responders available for each alert.

Word count: 4024

For peer review only - http://bmjopen.bmj.com/site/about/guidelines.xhtml

Author contributions: All authors developed the study protocol and design of participant information sheets, consent forms and topic guide. CMS recruited, conducted interviews and transcribed. CMS and FG analysed interview data. CMS drafted the manuscript, which all authors reviewed and edited.

Funding statement: This study/project is funded by the National Institute for Health Research (NIHR) Doctoral Research Fellowship (DRF 2017-10-095). The views expressed are those of the author(s) and not necessarily those of the NIHR or the Department of Health and Social Care.

Competing interests. CMS is an NIHR Doctoral Research Fellow and this work was part of his PhD. He also has a volunteer role at the Resuscitation Council (UK). MW is co-founder and Medical Director of GoodSAM. GDP is a NIHR Senior Investigator and is supported by research grants from the NIHR, Resuscitation Council (UK) and the British Heart Foundation.

Acknowledgements: Deepti Bal from GoodSAM for assistance in identifying and contacting GoodSAM first-responders; and members of CMS' PhD advisory group – Claire Hawkes, Christopher Hartley-Sharpe, Julian Hague (PPI), John Long (PPI) – who provided guidance and review on the project protocol, topic guides and participant information sheets.

For peer review only

References

1. Hawkes C, Booth S, Ji C, Brace-McDonnell SJ, *et al.* Epidemiology and outcomes from out-of-hospital cardiac arrests in England. *Resuscitation* 2017;**110**:133-40. doi:10.1016/j.resuscitation.2016.10.030
2. Holmberg MJ, Vognsen M, Andersen MS, *et al*. Bystander automated external defibrillator use and clinical outcomes after out-of-hospital cardiac arrest: A systematic review and meta-analysis. *Resuscitation* 2017;**120**:77-87. doi: 10.1016/j.resuscitation.2017.09.003
3. Smith CM, Lim Choi Keung SN, Khan MO, *et al.* Barriers and facilitators to public access defibrillation in out-of-hospital cardiac arrest: a systematic review. *Eur Heart J Qual Care Clin Outcomes* 2017;**3**:264-73. doi: 10.1093/ehjqcco/qcx023
4. Perkins GD, Travers AH, Berg RA, *et al.* Part 3: Adult basic life support and automated external defibrillation: 2015 International Consensus on Cardiopulmonary Resuscitation and Emergency Cardiovascular Care Science with Treatment Recommendations. *Resuscitation* 2015;**95**:e43-69. doi: 10.1016/j.resuscitation.2015.07.041
5. Pijls RWM, Nelemans PJ, Rahel BM, Gorgels APM. A text message alert system for trained volunteers improves out-of-hospital cardiac arrest survival. *Resuscitation* 2016;**105**:182-7. doi: 10.1016/j.resuscitation.2016.06.006
6. Ringh M, Rosenqvist M, Hollenberg J, *et al.* Mobile-Phone Dispatch of Laypersons for CPR in Out-of-Hospital Cardiac Arrest. *N Engl J Med* 2015;**372**:2316-25. doi: 10.1056/NEJMoa1406038
7. Brooks SC, Simmons G, Worthington H, *et al.* The PulsePoint Respond mobile device application to crowdsource basic life support for patients with out-of-hospital cardiac arrest: Challenges for optimal implementation. *Resuscitation* 2016;**98**:20-6. doi: 10.1016/j.resuscitation.2015.09.392
8. Smith CM, Wilson MH, Ghorbangholi A, *et al.* The use of trained volunteers in the response to out-of-hospital cardiac arrest - the GoodSAM experience. *Resuscitation* 2017;**121**:123-6. doi: 10.1016/j.resuscitation.2017.10.020
9. Barry T, Doheny MC, Masterson S, *et al.* Community first responders for out-of-hospital cardiac arrest in adults and children. *Cochrane Database Syst Rev* 2019;**7**:CD012764. doi: 10.1002/14651858.CD012764.pub2
10. Zijlstra JA, Stieglis R, Riedijk F, *et al.* Local lay rescuers with AEDs, alerted by text messages, contribute to early defibrillation in a Dutch out-of-hospital cardiac arrest dispatch system. *Resuscitation* 2014;**85**:1444-9. doi: 10.1016/j.resuscitation.2014.07.020
11. Ringh M, Fredman D, Nordberg P, *et al.* Mobile phone technology identifies and recruits trained citizens to perform CPR on out-of-hospital cardiac arrest victims prior to ambulance arrival. *Resuscitation* 2011;**82**:1514-8. doi: 10.1016/j.resuscitation.2011.07.033
12. Peberdy MA, Ottingham LV, Groh WJ, *et al.* Adverse events associated with lay emergency response programs: the public access defibrillation trial experience. *Resuscitation* 2006;**70**:59-65. doi: 10.1016/j.resuscitation.2005.10.030
13. Mathiesen WT, Bjorshol CA, Braut GS, Soreide E. Reactions and coping strategies in lay rescuers who have provided CPR to out-of-hospital cardiac arrest victims: a qualitative study. *BMJ Open* 2016;**6**:e010671. doi: 10.1136/bmjopen-2015-010671
14. Zijlstra JA, Beesems SG, De Haan RJ, Koster RW. Psychological impact on dispatched local lay rescuers performing bystander cardiopulmonary resuscitation. *Resuscitation* 2015;**92**:115-21. doi: 10.1016/j.resuscitation.2015.04.028
15. National Institutes of Health (June 14th 2019). The Scandinavian AED and Mobile Bystander Activation Trial (SAMBA). Available from: https://clinicaltrials.gov/ct2/show/NCT02992873 [Last accessed 3rd October 2019].
16. Atkins L, Francis J, Islam R, *et al.* A guide to using the Theoretical Domains Framework of behaviour change to investigate implementation problems. *Implement Sci* 2017;**12**:77.doi: 10.1186/s13012-017-0605-9

For peer review only - http://bmjopen.bmj.com/site/about/guidelines.xhtml

17. Michie S. Making psychological theory useful for implementing evidence based practice: a consensus approach. *Qual Saf Health Care* 2005;**14**:26-33. doi: 10.1136/qshc.2004.011155
18. Cane J, O'Connor D, Michie S. Validation of the theoretical domains framework for use in behaviour change and implementation research. *Implement Sci* 2012;**7**:37. doi: 10.1186/1748-5908-7-37
19. Michie S, Atkins L, West R. The Behaviour Change Wheel: A Guide To Designing Interventions. London: Silverback Publishing, 2014. ISBN: 978-1291846058
20. Francis JJ, Johnston M, Robertson C, *et al.* What is an adequate sample size? Operationalising data saturation for theory-based interview studies. *Psychol Health* 2010;**25**:1229-45. doi: 10.1080/08870440903194015
21. Mason M. Sample Size and Saturation in PhD Studies Using Qualitative Interviews. Forum: Qualitative Social Research. 2010;**11**(3). doi: http://dx.doi.org/10.17169/fqs-11.3.1428
22. Michie S, Richardson M, Johnston M, *et al.* The behavior change technique taxonomy (v1) of 93 hierarchically clustered techniques: building an international consensus for the reporting of behavior change interventions. *Ann Behav Med* 2013;**46**:81-95. doi: 10.1007/s12160-013-9486-6
23. O'Brien BC, Harris IB, Beckman TJ, *et al.* Standards for Reporting Qualitative Research: A Synthesis of Recommendations. *Acad Med* 2014;**89**:1245-51. doi: 10.1097/ACM.0000000000000388
24. Tourangeau R, Yan T. Sensitive questions in surveys. *Psychol Bull* 2007;**133**:859-83.doi: 10.1037/0033-2909.133.5.859
25. Britten N. *Qualitative Interviews*. In: Pope C, Mays N, editors. Qualitative Research in Health Care. 3rd ed. Oxford: Blackwell Publishing, 2006. p. 12-20. ISBN: 978-1405135122
26. Ormston R, Spencer L, Bernard M, Snape D. *The Foundations of Qualitative Research.* In: Ritchie J, Lewis J, McNaughton Nicholls C, Ormston R, editors. Qualitative Research Practice: A Guide for Social Science Students and Researchers. 2nd ed. London: SAGE, 2014. p. 1-25. ISBN: 978-1446209127
27. London Ambulance Service NHS Trust (August 2018). Taxi drivers get life-saving training for ambulance pilot scheme. Available from: https://www.londonambulance.nhs.uk/2018/08/01/taxi-drivers-get-life-saving-training-for-ambulance-pilot-scheme/ [last accessed 3rd October 2019].
28. Boutilier J, Brooks S, Janmohamed A, *et al.* Optimizing a Drone Network to Deliver Automated External Defibrillators. *Circulation* 2017;**135**:2454-65. doi: 10.1161/CIRCULATIONAHA.116.026318
29. Claesson A, Bäckman A, Ringh M, *et al.* Time to Delivery of an Automated External Defibrillator Using a Drone for Simulated Out-of-Hospital Cardiac Arrests vs Emergency Medical Services. *JAMA* 2017;**317**:2332-4. doi: 10.1001/jama.2017.3957

For peer review only - http://bmjopen.bmj.com/site/about/guidelines.xhtml

Figure 1: Possible barriers and facilitators to AED use – capability

Facilitators
Previous training in CPR/AED use
Previous real-life experience in CPR/AED use
Good awareness of Public Access Defibrillation
Using the app to check AED locations before an alert
Most participants felt capable and competent to respond to alerts
Barriers
Being less familiar of AED locations in unfamiliar areas
Varying recall of information given during an actual alert
Not recalling whether or not AED location were displayed at time of the alert
Not even considering AED retrieval at the time of the alert

Figure 2: Possible barriers and facilitators to AED use – opportunity

Facilitators
Having one’s own AED (Some first-responders did and had taken them to the scene)
Previously known AED available nearby
AED already present on scene
Barriers
Public-access AEDs
Perceived to be too far away from first-responder or patient at time of alert
Perceived difficulty finding exact location
Less availability during out-of-hours alerts
Perceived to be inaccessible in locked cabinets
App-specific issues:
GoodSAM location inaccurate at time of alert
Alert siren not always heard
App slow to display incident location once alert accepted
Healthcare professionals unable to leave patients at work to respond
Non-healthcare professionals feeling unable to leave work or dependents
Arriving after the ambulance service

For peer review only - http://bmjopen.bmj.com/site/about/guidelines.xhtml

Figure 3: Possible barriers and facilitators to AED use – motivation

Facilitators

Belief in the GoodSAM project and its benefits to patients
Debrief and follow-up after an alert
Knowledge that other first-responders were responding
Belief in the importance of AED use to patient survival
Planning ahead: finding AED locations
Confidence in AED use: helped by previous training and experience
Sense of professional duty

Barriers

Concerns about managing scene
Concerns about managing non-cardiac arrest patients
- Duty of care concerns
- Less confidence in abilities

Reduced motivation because of experience during recent alert(s):
- Too far from incident
- Belief that ambulance service would arrive before them
- Arriving after the ambulance service, even if accepting alert promptly

Being less likely to respond overnight
Prioritising arrival of scene and starting CPR as soon as possible over AED use
Not knowing if someone else was providing CPR at scene
Perceived difficulties in negotiation for AED release from its owner / custodian
Uncertainty about correct strategy: retrieve AED first or go direct to patient?
Difficulty explaining need to use AED to other bystanders
Concerns about acting outside normal sphere of work (healthcare professionals)

For peer review only

For peer review only - http://bmjopen.bmj.com/site/about/guidelines.xhtml

What needs to change (behavioural determinants)	Potential intervention	Intervention function	Policy category	Potential behavioural change techniques	Mode of delivery
Have the physical skills to use an AED effectively **(Capability)**	Deliver digital CPR/AED training	Training	Service provision: provide a specific training resource<br>Regulation: GoodSAM could require completion of training for continued registration with app	Demonstration of behaviour<br>Feedback on behaviour<br>Behavioural practice / rehearsal	Online or in-app training (e.g. interactive video-training package 'Lifesaver' (https://life-saver.org.uk)
Know about the existence of public-access AEDs **(Capability)**	Provide reminders about nearby AED locations	Education	Communication / marketing: provide regular reminders about AED locations<br>Guidelines: introduce recommendations about checking for AED locations into GoodSAM code of conduct	Prompts / cues	Information via e-mail<br>Visual prompt in-app<br>Audio / voice prompt in-app<br>*Delivered at regular, spaced intervals – **not** at the time of an alert*
Be aware of nearby public-access AED locations **(Opportunity)**	Highlight the location of the nearest AED at the time of the alert	Enablement	Environmental / Social planning: designing the in-app environment to enable people to recognise the location of the nearest AED	Prompts / cues	Visual prompt in-app<br>Audio / voice prompt in-app<br>*Delivered during an alert*
Be able to locate and retrieve an AED that is close enough and accessible **(Opportunity)**	Provide access codes to AEDs in locked cabinets	Environmental Restructuring	Guidelines: Recommendations about how to access public-access AEDs<br>Regulation: Voluntary agreement with ambulance services and other AED custodians to provide access codes<br>Legislation: National-level laws to facilitate access to locked cabinets, e.g. mandated provision of access codes	Prompts / cues	Visual prompt in-app<br>Audio / voice prompt in-app<br>*Delivered during an alert, specific to relevant and local AEDs only*

For peer review only - http://bmjopen.bmj.com/site/about/guidelines.xhtml

Be able to locate and retrieve an AED that is close enough and accessible **(Opportunity)**	Provide standardised information to show custodians of public-access AEDs	Enablement	Guidelines: provide a document to GoodSAM first-responders with recommended form of words<br>Communication / marketing: information campaign targeting AED custodians	Information about health consequences<br>Salience of consequences<br>Information about others' approval<br>(Use of a) Credible source	Print media campaign<br>Digital media campaign<br>Printed card (to leave with custodian)<br>Visual display in-app (to show custodian)
Arrive at the patient before the ambulance service **(Opportunity)**	Streamline location and travel route information at the time of an alert	Enablement	Communication / marketing: deliver 'real-time' information to GoodSAM responder via the app<br>Environmental / social planning: designing in-app environment to enable recognition of AED location and incident	Prompts / cues	Visual prompt in-app<br>Audio / voice prompt in-app<br>*Delivered during an alert*
Have access to one's own AED **(Opportunity)**	Equip first-responders with their own AEDs	Enablement	Regulation: rules designating which GoodSAM responders should be equipped with an AED and how they should respond with it<br>Guidelines: how and when to carry and respond with AED	Adding objects to environment<br>Action planning<br>Review behaviour goal<br>Review outcome goal	Information about appropriate use of AED delivered with equipment<br>Supplementary information via e-mail or via the app
Believe that it is appropriate to retrieve an AED **(Motivation)**	Provide reminders about appropriate use of public-access AEDs	Education	Communication / marketing: reminders re: appropriate AED use<br>Guidelines: recommendations about appropriate situations for AED use	Information about health consequences<br>Information about social and environmental consequences<br>Salience of consequences	Information via e-mail<br>Information via app<br>*Delivered at regular, spaced intervals – **not** at the time of an alert*

For peer review only - http://bmjopen.bmj.com/site/about/guidelines.xhtml

Believe that it is appropriate to retrieve an AED **(Motivation)**	Provide information about route and time to the nearest public-access AED(s)	Persuasion	Communication / marketing: deliver 'real-time' information to GoodSAM responder via app<br>Environmental / social planning: designing in-app environment to display AED and incident location	Information about social and environmental consequences	Visual prompt in-app<br>Audio / voice prompt in-app
Believe that it is appropriate to retrieve an AED **(Motivation)**	Send some responders (if multiple responders available) to retrieve a public-access AED first	Persuasion	Communication / marketing: sharing information about each responders' actions (so all involved know that someone is retrieving AED and someone is going to scene)<br>Guidelines: explaining why GoodSAM responder(s) should retrieve AED first when asked<br>Regulation: rules on when GoodSAM responder(s) should retrieve AED first	Information about social and environmental consequences	Website Code of Conduct<br>E-mail updates<br>*Delivered at regular, spaced intervals –* ***not*** *at the time of an alert*<br><br>Visual prompt in-app<br>Audio / voice prompt in-app<br>*Delivered during an alert*
Believe that one can retrieve an AED and reach patient before the ambulance service **(Motivation)**	Provide time-to-patient and ambulance response-time estimates at the time of an alert	Persuasion	Communication / marketing: deliver 'real-time' information to GoodSAM responder via app<br>Regulation: organisational rules about how to safely obtain this information from ambulance services and provide it to GoodSAM responders	Information about social and environmental consequences<br>Salience of consequences<br>Credible source	Visual prompt in-app<br>Audio / voice prompt in-app<br>*Delivered during an alert*

For peer review only - http://bmjopen.bmj.com/site/about/guidelines.xhtml

Be confident to use an AED effectively **(Motivation)**	Deliver motivational messages regarding AED use	Persuasion	Communication / marketing: deliver motivational information directly to responder at time of alert	[Verbal] persuasion about capability	Visual prompt in-app<br>Audio / voice prompt in-app<br>*Delivered during an alert*
Overcome anxiety and stress about responding or using an AED **(Motivation)**	Offer voluntary debrief after an alert (to increase AED use and/or future response rate)	Enablement	Service provision: survey GoodSAM responders, identify those at risk of psychological harm, and offer appropriate follow-up for those who need it	Social support (emotional)<br>Review behaviour goals<br>Review outcome goals	In-app survey post-alert<br>Face-to-face / telephone follow-up if needed

Table 1: Potential interventions to increase AED use in GoodSAM first-responders

For peer review only - http://bmjopen.bmj.com/site/about/guidelines.xhtml

DEFIBRILLATOR USE

BY VOLUNTEER FIRST-RESPONDERS FOR OUT-OF-HOSPITAL CARDIAC ARREST

BACKGROUND:

GoodSAM

Ambulance services can use **GoodSAM** to alert volunteer first-responders to a nearby cardiac arrest

The app provides directions to the patient, and shows nearby public-access defibrillators

QUESTION

Do **GoodSAM** first-responders use defibrillators when alerted? What are the barriers?

METHODS:

Telephone interviews with **30** **GoodSAM** first-responders shortly after an alert

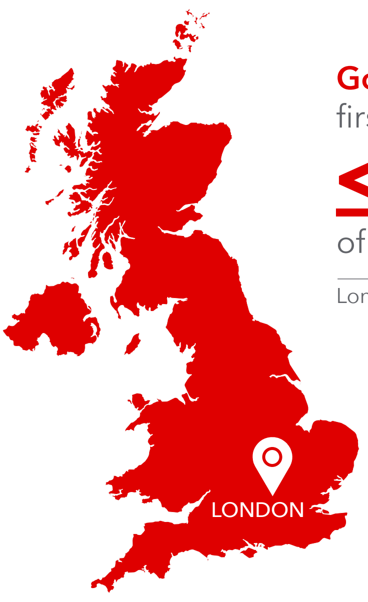

GoodSAM first-responders alerted if **≤300m** of an incident

London, July and November 2018

RESULTS:

14/30 accepted the alert

1 retrieved a public-access defibrillator

3 others took their own defibrillators

We used the **Capability, Opportunity, Motivation Behaviour (COM-B) model** to categorise barriers to defibrillator use

CAPABILITY

Responders were capable of using defibrillators

Didn't think about using them at time of alert

OPPORTUNITY

Defibrillators not close enough to retrieve during an alert

Defibrillators not accessible out-of-hours

MOTIVATION

Responders believed in the benefit of defibrillators

Responders were concerned about delaying CPR by retrieving a defibrillator on the way

We used the **Behaviour Change Wheel (BCW)** to find possible ways of improving defibrillator use, including:

- Highlighting the position of the nearest defibrillator during an alert
- Providing travel routes and times to patient via nearest defibrillator
- Provide access codes to locked defibrillator cabinets
- Equipping volunteers with their own defibrillator

GoodSAM

Higher first-responder density would enable strategies to send some directly to the patient and others to retrieve a defibrillator on the way

For peer review only
For peer review only - http://bmjopen.bmj.com/site/about/guidelines.xhtml

		Reporting Item	Page Number
**Title**			
	#1	Concise description of the nature and topic of the study identifying the study as qualitative or indicating the approach (e.g. ethnography, grounded theory) or data collection methods (e.g. interview, focus group) is recommended	1
**Abstract**			
	#2	Summary of the key elements of the study using the abstract format of the intended publication; typically includes background, purpose, methods, results and conclusions	2-3
**Introduction**			
Problem formulation	#3	Description and signifcance of the problem / phenomenon studied: review of relevant theory and empirical work; problem statement	5-7
Purpose or research question	#4	Purpose of the study and specific objectives or questions	7
**Methods**			
Qualitative approach and research paradigm	#5	Qualitative approach (e.g. ethnography, grounded theory, case study, phenomenolgy,	8-11

For peer review only

		narrative research) and guiding theory if appropriate; identifying the research paradigm (e.g. postpositivist, constructivist / interpretivist) is also recommended; rationale. The rationale should briefly discuss the justification for choosing that theory, approach, method or technique rather than other options available; the assumptions and limitations implicit in those choices and how those choices influence study conclusions and transferability. As appropriate the rationale for several items might be discussed together.	
Researcher characteristics and reflexivity	#6	Researchers' characteristics that may influence the research, including personal attributes, qualifications / experience, relationship with participants, assumptions and / or presuppositions; potential or actual interaction between researchers' characteristics and the research questions, approach, methods, results and / or transferability	In Discussion (Strengths and Weaknesses) 22
Context	#7	Setting / site and salient contextual factors; rationale	8
Sampling strategy	#8	How and why research participants, documents, or events were selected; criteria for deciding	9

For peer review only

		when no further sampling was necessary (e.g. sampling saturation); rationale	
Ethical issues pertaining to human subjects	#9	Documentation of approval by an appropriate ethics review board and participant consent, or explanation for lack thereof; other confidentiality and data security issues	12
Data collection methods	#10	Types of data collected; details of data collection procedures including (as appropriate) start and stop dates of data collection and analysis, iterative process, triangulation of sources / methods, and modification of procedures in response to evolving study findings; rationale	9-11
Data collection instruments and technologies	#11	Description of instruments (e.g. interview guides, questionnaires) and devices (e.g. audio recorders) used for data collection; if / how the instruments(s) changed over the course of the study	9
Units of study	#12	Number and relevant characteristics of participants, documents, or events included in the study; level of participation (could be reported in results)	12 (reported in results)
Data processing	#13	Methods for processing data prior to and during analysis, including transcription, data entry, data management and security, verification of data	10-11

For peer review only

		integrity, data coding, and anonymisation / deidentification of excerpts	
Data analysis	#14	Process by which inferences, themes, etc. were identified and developed, including the researchers involved in data analysis; usually references a specific paradigm or approach; rationale	11
Techniques to enhance trustworthiness	#15	Techniques to enhance trustworthiness and credibility of data analysis (e.g. member checking, audit trail, triangulation); rationale	11
**Results/findings**			
Syntheses and interpretation	#16	Main findings (e.g. interpretations, inferences, and themes); might include development of a theory or model, or integration with prior research or theory	12-20, Figures 1-3, Table 1
Links to empirical data	#17	Evidence (e.g. quotes, field notes, text excerpts, photographs) to substantiate analytic findings	12-20
**Discussion**			
Intergration with prior work, implications, transferability and contribution(s) to the field	#18	Short summary of main findings; explanation of how findings and conclusions connect to, support, elaborate on, or challenge conclusions of earlier scholarship; discussion of scope of application / generalizability; identification of	21, 22-25

For peer review only

		unique contributions(s) to scholarship in a discipline or field	
Limitations	#19	Trustworthiness and limitations of findings	Box on page 4, 22
**Other**			
Conflicts of interest	#20	Potential sources of influence of perceived influence on study conduct and conclusions; how these were managed	26
Funding	#21	Sources of funding and other support; role of funders in data collection, interpretation and reporting	26

None The SRQR checklist is distributed with permission of Wolters Kluwer © 2014 by the Association of American Medical Colleges. This checklist can be completed online using https://www.goodreports.org/, a tool made by the EQUATOR Network in collaboration with Penelope.ai

For peer review only

BMJ Open

Identifying and overcoming barriers to Automated External Defibrillator use by GoodSAM volunteer first-responders in out-of-hospital cardiac arrest using the Theoretical Domains Framework and Behaviour Change Wheel. A qualitative study.

Journal:	*BMJ Open*
Manuscript ID	bmjopen-2019-034908.R1
Article Type:	Original research
Date Submitted by the Author:	03-Feb-2020
Complete List of Authors:	Smith, Christopher Matthew; University of Warwick, Warwick Clinical Trials Unit<br>Griffiths, Frances; University of Warwick, Warwick Medical School<br>Fothergill, Rachael; London Ambulance Service NHS Trust, Clinical Audit and Research Unit<br>Vlaev, Ivo; Warwick Business School,<br>Perkins, Gavin; University of Warwick, Warwick Clinical Trials Unit
**Primary Subject Heading**:	Emergency medicine
Secondary Subject Heading:	Public health, Qualitative research
Keywords:	QUALITATIVE RESEARCH, PUBLIC HEALTH, ACCIDENT & EMERGENCY MEDICINE

SCHOLARONE™
Manuscripts

I, the Submitting Author has the right to grant and does grant on behalf of all authors of the Work (as defined in the below author licence), an exclusive licence and/or a non-exclusive licence for contributions from authors who are: i) UK Crown employees; ii) where BMJ has agreed a CC-BY licence shall apply, and/or iii) in accordance with the terms applicable for US Federal Government officers or employees acting as part of their official duties; on a worldwide, perpetual, irrevocable, royalty-free basis to BMJ Publishing Group Ltd ("BMJ") its licensees and where the relevant Journal is co-owned by BMJ to the co-owners of the Journal, to publish the Work in this journal and any other BMJ products and to exploit all rights, as set out in our licence.

The Submitting Author accepts and understands that any supply made under these terms is made by BMJ to the Submitting Author unless you are acting as an employee on behalf of your employer or a postgraduate student of an affiliated institution which is paying any applicable article publishing charge ("APC") for Open Access articles. Where the Submitting Author wishes to make the Work available on an Open Access basis (and intends to pay the relevant APC), the terms of reuse of such Open Access shall be governed by a Creative Commons licence – details of these licences and which Creative Commons licence will apply to this Work are set out in our licence referred to above.

Other than as permitted in any relevant BMJ Author's Self Archiving Policies, I confirm this Work has not been accepted for publication elsewhere, is not being considered for publication elsewhere and does not duplicate material already published. I confirm all authors consent to publication of this Work and authorise the granting of this licence.

For peer review only

Identifying and overcoming barriers to Automated External Defibrillator use by GoodSAM volunteer first-responders in out-of-hospital cardiac arrest using the Theoretical Domains Framework and Behaviour Change Wheel. A qualitative study.

Christopher M Smith [1] (corresponding author: c.smith.20@warwick.ac.uk)

Frances Griffiths [2]

Rachael T Fothergill [3]

Ivo Vlaev [4]

Gavin D Perkins [1]

[1] Warwick Clinical Trials Unit, University of Warwick, Coventry, CV4 7AL, UK

[2] Division of Health Sciences, Warwick Medical School, University of Warwick, Coventry, CV4 7AL, UK

[3] Clinical Audit and Research Unit, London Ambulance Service NHS Trust, London, SE1 0BW

[4] Warwick Business School, University of Warwick, Coventry, CV4 7AL, UK

Keywords: Out-Of-Hospital Cardiac Arrest; defibrillators; cardiopulmonary resuscitation; qualitative research

For peer review only

Abstract

Objectives: GoodSAM is a mobile-phone app that integrates with UK ambulance services. During a 999 call, if a call-handler diagnoses cardiac arrest, nearby volunteer first-responders registered with the app are alerted. They can give CPR and/or use a public-access Automated External Defibrillator (AED). We aimed to identify means of increasing AED use by GoodSAM first-responders.

Methods: We conducted semi-structured telephone interviews, using the Theoretical Domains Framework to identify and classify barriers to AED use. We analysed findings using the Capability, Opportunity, Motivation, Behaviour (COM-B) model and subsequently used the Behaviour Change Wheel to develop potential interventions to improve AED use.

Setting: London, UK.

Participants: GoodSAM first-responders alerted in the previous seven days about a cardiac arrest.

Results: We conducted 30 telephone interviews in two batches in July and October 2018. A public-access AED was taken to scene once, one had already been attached on scene another time, and three participants took their own AEDs when responding. Most first-responders felt capable and motivated to use public-access AEDs, but were concerned about delaying CPR if they retrieved one, and frustrated when arriving after the ambulance service. They perceived lack of opportunities due to unavailable and inaccessible AEDs, particularly out-of-hours. We subsequently developed thirteen potential interventions to increase AED use, for future testing.

Conclusions: GoodSAM first-responders used AEDs occasionally, despite a capability and motivation to do so. Those operating volunteer first-responder systems

For peer review only - http://bmjopen.bmj.com/site/about/guidelines.xhtml

should consider our proposed interventions to improve AED use. Of particular clinical importance are: highlighting AED location; and providing route/time estimates to the patient via the nearest AED. This would help single responders make appropriate decisions about AED retrieval. As AED collection may extend time to reach the patient, where there is sufficient density of potential responders, systems could send one responder to initiate CPR and another to collect an AED.

Abstract word count: 300

For peer review only

For peer review only - http://bmjopen.bmj.com/site/about/guidelines.xhtml

Strengths and limitations of this study

- This interview study is the first time behaviours affecting Automated External Defibrillator (AED) use of volunteer first-responders activated to out-of-hospital cardiac arrest have been explored using the Theoretical Domains Framework and Behaviour Change Wheel
- We have developed a list of potential interventions to increase AED use that can be tested by *any* similar first-responder system, of which there are a number worldwide
- Actual AED use in this study was infrequent: interview responses focus on intended actions and may not be representative of participants' behaviours in future time-critical emergency situations

review only

For peer review only - http://bmjopen.bmj.com/site/about/guidelines.xhtml

Introduction

Fewer than one in ten patients who suffer an out-of-hospital cardiac arrest (OHCA) survive to hospital discharge in England (1). Public Access Defibrillation (PAD) – bystander use of public-access Automated External Defibrillators (AEDs) – is associated with doubled survival to hospital discharge with good neurological function (2). However, PAD is used in fewer than 5% of OHCA (1,3).

Volunteer first-responder systems

Mobile app-based volunteer first-responder systems are one approach to overcome limited PAD use (4). A number of countries have app-based or text-message systems to alert bystanders to a nearby OHCA, including Netherlands (5), Sweden (6) and USA (7). 'GoodSAM' (https://www.goodsamapp.org/) is the system that operates in the UK (8). Patient outcome data are limited (9). The only randomised controlled trial, comparing a text-message alert system supplementing ambulance service care vs ambulance service care alone, reported significantly higher bystander CPR rates (primary outcome: 61.6% vs 47.8%, p<0.001) but no significant difference in the secondary outcome of 30-day survival (11.2% vs 8.6%, p=0.28). However, there was no mention of PAD in this system (6). In an observational study in the Netherlands (2012-2014), OHCA patients attended by text-message-alerted first-responders were 2.8 times more likely to survive to hospital discharge than those for whom alerted first-responders did not attend. First-responders attached an AED in 26.8% cases (5).

For peer review only - http://bmjopen.bmj.com/site/about/guidelines.xhtml

Barriers and facilitators to PAD

A systematic review considering barriers and facilitators to PAD reported that public-access AEDs are located close to few OHCA, and their accessibility is reduced out-of-hours. Knowledge, awareness and willingness to use public-access AED vary, but are increased by prior CPR/AED training (3). In volunteer first-responder systems, not reaching the scene after accepting an alert (5,7,10), not being the first to arrive and perform CPR (7,11) or attach an AED (10) have all been reported.

There is limited knowledge about the effect of first-responder systems and how to most effectively deploy AEDs within them, and recognition that we need high-quality research to investigate this (4). The Scandinavian AED and Mobile Bystander Activation (SAMBA) trial (12) is currently randomising volunteer first-responders using the 'Heartrunner' app in Denmark and Sweden to either: all responders travel direct to the patient or; some responders retrieve the nearest AED first. The primary outcome is the proportion of patients who have an AED attached before the ambulance service arrives.

Theoretical frameworks to develop interventions for increased PAD use

There is no work using a theoretically-informed behavioural framework to address low AED use in volunteer first-responder systems. The Theoretical Domains Framework (TDF) can be used to identify specific barriers or facilitators to a particular outcome (13,14). The fourteen domains of the revised version of the TDF (15) can be further grouped and integrated with the COM-B (Capability, Opportunity, Motivation)

For peer review only - http://bmjopen.bmj.com/site/about/guidelines.xhtml

behavioural framework. COM-B characterises three targets for behavioural change in order to inform the design of healthcare interventions and can, in turn, be linked to the Behaviour Change Wheel (BCW) (16). The BCW is used to develop interventions and the means of implementing them.

Research question

How can we increase the numbers of AEDs taken to an OHCA by a GoodSAM first-responder following an alert?

To answer this, we took data from an interview study and used the BCW to develop a theoretically-informed list of interventions to increase public-access AED use by GoodSAM first-responders during an alert.

For peer review only

For peer review only - http://bmjopen.bmj.com/site/about/guidelines.xhtml

Methods

Description of GoodSAM system

GoodSAM is an app-based system that integrates with several local ambulance services to alert trained volunteers to an OHCA (8). In the UK, GoodSAM first-responders require at least in-date CPR training to register with the app. A call-handler, using a Computer Aided Dispatch (CAD) system during a 999 call, allocates a code identifying the clinical problem. If this code indicates that a patient is suffering a current or imminent cardiac arrest, the system automatically activates GoodSAM. First-responders can accept or decline this alert. The app displays incident address, route and nearby public-access AEDs. They decide themselves whether to retrieve an AED or travel directly to scene: they receive no instruction via the app.

There were 298 OHCA in London in July 2018, and 306 in October 2018 (17). There are 3-5 GoodSAM notifications daily for OHCA. In July 2018, GoodSAM responders accepted 19% of alerts in London (information from GoodSAM).

Study setting

London, UK. GoodSAM integrated with London Ambulance Service in October 2015. Following an OHCA diagnosis during the 999 call, GoodSAM first-responders within 300m of the incident receive an alert siren on their mobile device.

For peer review only - http://bmjopen.bmj.com/site/about/guidelines.xhtml

Study design

Telephone semi-structured interviews performed with GoodSAM first-responders in London, with responses coded to TDF domains. We developed topic guides based on COM-B. The focus in first-responder interviews was on decisions to use a public-access AED.

Participant selection and recruitment

We approached first-responders consecutively in two blocks in July and October 2018, shortly after they received an alert in London. We made an a priori decision to conduct 30 interviews before assessing for data saturation – defined here as no new information emerging from interviews 28-30 (18). We felt this was pragmatic and consistent with other studies using qualitative interviews (19).

Interview procedure

We approached potential participants via e-mail, with a participant information sheet and consent statements attached. Interested participants replied to CMS, who subsequently arranged an interview. We sent no further e-mails to non-respondents.

CMS conducted interviews with GoodSAM first-responders using Microsoft Skype (audio only) to the telephone number provided, and recorded directly onto computer using QuickTime player. CMS introduced himself as a clinician but explained his current role as a University researcher undertaking a PhD. Interviewer and participants

For peer review only - http://bmjopen.bmj.com/site/about/guidelines.xhtml

did not previously know one another. Participants could ask questions before recording started. Recording began and participants gave verbal agreement to consent statements, which the interviewer read aloud. Interviews lasted a median of 14:56min (range 7:41-24:01), and recording continued until the call ended. All interviews took place at least 24 hours after the initial invitation, and within seven days of the GoodSAM alert.

We kept audio files and transcriptions in separate encrypted computer folders. No participant-identifying information was associated or stored with audio files or transcripts.

Interview data analysis

CMS transcribed and coded interviews using NVivo (QSR International). CMS and FG subjected the first three interviews to detailed line-by-line coding without reference to the TDF: they judged that the codes and emerging themes sufficiently matched with TDF domains for us to comprehensively code information to these TDF domains. CMS subsequently coded all interviews using the TDF, with periodic checking undertaken by FG and iterative update and review of coding as required. We remained alert to data that did not fit into a TDF domain. We matched coded material from TDF domains to components of COM-B, with which the TDF integrates (16). We subsequently analysed the data and synthesised a narrative about the first-responders' experiences relating to their capability (subdivided into physical and psychological), opportunity (social and physical) and motivation (automatic and reflective) to use an AED as part of their response.

For peer review only

Developing interventions

We developed potential interventions to improve AED use by GoodSAM first-responders, using a step-by-step approach described in the BCW. As described (16) we grouped and integrated TDF domains into the COM-B framework which, in turn, were linked to the BCW. Using the BCW, we identified relevant intervention functions for behavioural determinants that would have to change to increase AED use. We further assessed these intervention functions (from a list of nine in the BCW) using APEASE (Affordability, Practicability, Effectiveness/cost-effectiveness, Safety, Efficacy) criteria and identified policy categories, behavioural change techniques and modes of delivery. We selected behavioural change techniques described in the Behaviour Change Techniques Taxonomy (BCTT) version 1 (20): these associate with intervention functions in the BCW (16).

Ultimately, we decided that presenting our findings – initially coded to TDF domains – according to whether they represented capability, opportunity or motivation to change behaviour, was the most accessible way to communicate our findings.

Patient and Public Involvement

There were two lay representatives on the project's advisory committee. They contributed to the topic guide, participant information sheets, consent forms and protocol.

For peer review only - http://bmjopen.bmj.com/site/about/guidelines.xhtml

Ethics

The study received approval from University of Warwick Biomedical Sciences Research Ethics Committee on 16/03/2018 (REGO-2018-2164).

Reporting

We report this study according to Standards for Reporting Qualitative Research (21) (**supplementary file**)

For peer review only

For peer review only - http://bmjopen.bmj.com/site/about/guidelines.xhtml

Results

1. Interview Study

We conducted 30 telephone interviews, from 248 e-mail invitations. Twenty-one interviews took place in July 2018. After reviewing the topic guide we conducted the remaining interviews in October 2018. A higher proportion of our first twenty-one participants had accepted their latest alert (12/21, 57%) compared to the London average, so for these last nine interviews we invited only those who had declined their latest alert. We determined that we had achieved data saturation after 30 interviews.

Ultimately, 14/30 (47%) responders accepted their most recent alert. Six reached the patient's side; three before the ambulance service. Four of eleven patients, where known, were in cardiac arrest. Most participants had also received previous alerts so we also discussed issues relating to these alerts, starting with the most recent.

Eleven first-responders had previous healthcare experience. Overall figures for all GoodSAM first-responders were not available.

We present a summary of barriers and facilitators to AED use in **Figures 1-3.**

Physical capability

No participant said they felt unable to provide CPR or use an AED, and all who commented believed previous CPR/AED training facilitated CPR and AED use when

For peer review only - http://bmjopen.bmj.com/site/about/guidelines.xhtml

responding. Eight participants reported that real-life experience of OHCA would help them effectively use an AED in future responses.

Psychological capability

Participants were knowledgeable about public-access AEDs, likely locations and that the app displayed them:

> "I'm aware that tube stations, Pret, the usual kind of suspects will have them, so there's quite a few within close proximity, not on my route, but if I needed help I could direct someone, go to just up the street, go to Boots" (#10)

Three participants reported knowledge of AEDs on the app that were unavailable out-of-hours (#12,#14,#24). Six participants knew about an AED's location only after browsing the app (i.e. when *not* receiving an alert). Four participants said they were less knowledgeable about AED locations outside their home area:

> "Most places I wouldn't [know where an AED was] unless I looked on the GoodSAM app and it was on the map." (#20)

Nine reported not remembering whether the app displayed a nearby AED at the time of the alert; seven others reported that they remembered seeing an AED when alerted.

For peer review only

Social opportunity

One participant (who had declined every alert that he had received) did not feel pressured to act by other bystanders, as the patient was somewhere else:

> "It's easier to be influenced I think by a lack of confidence if it's not a real human being in front of you." (#21)

Physical opportunity

No participant reported problems accessing the patient when they were first on-scene. However, eleven participants arrived after the ambulance service on their most recent alert. Three participants (#14;#17;#25) reported this on earlier alerts as well.

Nine participants reported on their interaction with ambulance service personnel. Five participants (#5;#16;#22;#23;#24) reported helping on scene:

> "Once he realised the level of training that I have he was very excited about the fact that I was there." (#22)

However, three participants (#15;#17;#23) reported occasions when the ambulance service did not accept assistance. Two participants (#14;#23) additionally reported that ambulance personnel were cautious about letting them help, particularly without identification.

For peer review only - http://bmjopen.bmj.com/site/about/guidelines.xhtml

Five participants felt that a standard means of identifying oneself as a first-responder would be useful. Seven participants were concerned about how they would find and negotiate with an AED's custodian for its removal to a different area:

> "There's a whole rigmarole, can I have a defibrillator, I'm a GoodSAM volunteer, are these people going to know?" (#17)

Participant 21 expressed a contrasting view:

> "I kind of imagine if ever an organisation or a building or whatever has an AED and you ran in, you said somebody's having a cardiac arrest can I borrow your AED, I don't imagine that many people would say no." (#21)

A public-access AED was taken to a patient once when considering participants' most recent alerts, by participant#3, who obtained it from their workplace. Participant#30 found an AED already attached to the patient on-scene. Three participants (#6;#13;#16) took their own AEDs to the scene during their most recent alert. Eight participants reported that there were no public-access AEDs close enough to retrieve when they accepted an alert.

Two other participants (#26;#30) believed that finding an AED's exact location would be difficult. Participant 26 was unsure how one would retrieve an AED that was kept in a code-locked cabinet (which many are), and whether or not this code would be available through the app. Contrastingly, two participants (#5;#13) believed that AEDs would not be too difficult to find:

For peer review only - http://bmjopen.bmj.com/site/about/guidelines.xhtml

For peer review only

"I think most of them are fairly easy accessible, I think they generally have to be in well-located places and easy to find." (#13)

Four participants commented on there being fewer public-access AEDs available out-of-hours:

"They're in GP surgeries or local shops and both of the times I've responded they've been out-of-hours." (#12)

Automatic motivation

Seven participants reported anxiety about on-scene management, including dealing with patients who have not suffered a cardiac arrest:

"I suppose if there was like mental health issues, or drugs and alcohol involved, I would be a bit apprehensive like getting involved in that" (#6)

Three participants (#8;#14;#23) expressed concern about what had happened to a patient when they did not accept an alert:

"I did have that real nagging feeling afterwards about 'oh my god, you know, that person could've died because I didn't go" (#14)

For peer review only - http://bmjopen.bmj.com/site/about/guidelines.xhtml

Two participants (#6;#28) said that they would welcome incident feedback, or a "debrief" from the ambulance service (#9).

Reflective motivation

Nine participants said that GoodSAM was an important initiative. Three (#6;#9,#16) expressed the belief that it has already saved lives.

Several factors affected or would affect the intention to accept an alert. Eleven participants cited distance to the incident. Six participants reported a belief that ambulance personnel would arrive before them, reinforced by previous experience:

> "I find that very frustrating, because you drop what you're doing, go and assist someone and then by the time you get there there's already enough people so you just kind of not go." (#14)

One participant (#30) was informed by the app about other GoodSAM first-responders accepting or declining the same alert, but did not believe this had affected the decision to respond. Twenty participants stated a preference for going directly to the patient to assess the situation and provide CPR, rather than retrieving an AED first:

> "[An AED] obviously makes a massive difference to early survival, but I think I would deem somebody doing good-quality chest compressions as a higher priority than taking an extra five to ten minutes to find the local machine." (#26)

For peer review only - http://bmjopen.bmj.com/site/about/guidelines.xhtml

Gaining access to an AED from its custodian might also affect the motivation to retrieve one:

> "I wouldn't waste too much time… If I couldn't get it immediately within 5 to 10 seconds I would be on my way without it." (#19)

Seven participants commented on the importance of early AED use to survival, and three (#10;#18;#20) said they would look for an AED first if possible:

> "That's the best way to save their life really. It would be worth the extra minute to get the defibrillator first." (#18)

Three participants (#13;#17;#21) talked about the uncertainty about whether or not to retrieve an AED first:

> "I appreciate obviously getting an early shock as quick as possible is preferable for the patient but equally I would feel bad if there was someone not doing any compressions and I had to spend five minutes trying to find a defib." (#13)

Thirteen participants reported that an AED's proximity to the patient or the route taken to the scene would affect their intention to retrieve it. Five participants said they had prepared by finding AED locations on the GoodSAM app before receiving an alert.

For peer review only

For peer review only - http://bmjopen.bmj.com/site/about/guidelines.xhtml

Seventeen participants explicitly expressed confidence in AED use. Reasons given were previous training (#15;#18), previous experience (#3;#6;#10;#11) and perceived ease of use (#11;#15;#18). On-scene issues could affect AED use:

> "Yeah I would be able to do that [use an AED], I think 90%. My hesitancy would be about dealing with the patient and the people around them. To suddenly say right we've got to get this top off and I need to connect these things straight away… So, let's say 80% confident." (#9)

Three participants (#22;#24;#28) remarked that their sense of professional duty affected their motivation to respond to a GoodSAM alert:

> "It's somewhat a large portion of what I do and who I am, so there's not many situations where I wouldn't want to respond to." (#24)

However, three responders (#5,#22,#23 – all healthcare professionals) stated concern about intervening outside their usual environment:

> "It was a little bit more nerve-wracking because you walk in and you're like I want to do this, this and this but actually I can only do CPR." (#5)

For peer review only - http://bmjopen.bmj.com/site/about/guidelines.xhtml

2. Developing interventions to improve AED use by GoodSAM first-responders

We identified ten behavioural determinants that needed to change to increase AED use, thirteen potential interventions to achieve this, the behavioural change techniques required to implement them and how to deliver them **(Table 1)**.

Potential interventions were: delivering digital CPR/AED training; providing reminders about nearby AED locations; highlighting the location of the nearest AED at the time of an alert; providing access codes to AEDs in locked cabinets; providing standardised information to show AED custodians when negotiating for an AED's release; streamlining incident location and travel route information provided at the time of an alert; equipping GoodSAM first-responders with their own AEDs; providing reminders about the appropriate use of AEDs; providing distance and time estimates, if travelling to the patient via the nearest AED, at the time of the alert; sending some responders directly to the patient and some via a nearby AED; providing time-to-patient (for first-responder) and ambulance response-time estimates at the time of the alert; delivering motivational messages related to AED use; offering voluntary debrief after an alert.

We have also produced an infographic (**supplementary file**) explaining the study and its main findings.

For peer review only - http://bmjopen.bmj.com/site/about/guidelines.xhtml

Discussion

Principal findings

We identified thirteen interventions that might increase AED use. Two are primarily concerned with increasing capability, five with increasing opportunity and six with increasing motivation. Some of these interventions also concern means of increasing acceptance of alerts. The two are inexorably linked: there can be no AED use if the first-responder has declined the alert.

Our findings suggest a potential to improve AED use during a GoodSAM response, and these may be relevant to other volunteer first-responder systems. In the interview study, one GoodSAM first-responder retrieved a public-access AED after an alert. In almost all cases participants reported a capability and motivation to provide CPR and use an AED. Whilst knowledgeable about PAD, they saw location and accessibility (particularly out-of-hours) of public-access AEDs as barriers to use. There was concern about the time required to retrieve an AED and not knowing if bystanders were performing CPR during this time. Participants also used the app to familiarise themselves with location and access hours for public-access AEDs. Many reached the patient after the ambulance service and this could affect motivation to respond to future alerts.

For peer review only - http://bmjopen.bmj.com/site/about/guidelines.xhtml

Comparison with the literature

We have presented a number of novel findings. In addition, this study support previous findings about the lack of appropriately placed AEDs, particularly out-of-hours (3). Our participants reported about not arriving first on scene and/or not intervening in an OHCA, in similarity with reports from other volunteer first-responder systems (7,10,11). Volunteer first-responders in a Dutch system also reported stress, although this had resolved (in 81%) or mild (19%) by four to six weeks (22).

Implications for clinicians and policymakers

This research suggests that even capable and motivated volunteer first-responders rarely use public-access AEDs. We can reduce physical restraints to AED retrieval by means such as highlighting the location of AEDs, providing access codes to locked cabinets and displaying route distance and time estimates to the nearest AED and to the patient. However, a key concern about diverting to the nearest AED may always be that this increases the length of time that an OHCA patient does not receive CPR. The decision about the appropriateness of such a strategy remains that of the first-responder at the time of the alert.

A key goal when establishing GoodSAM in London was the provision of early CPR, rather than a specific focus on diverting GoodSAM first-responders to retrieve an AED. Patients will not always be in cardiac arrest (four of eleven were in this study, where known) and responders will not always arrive first. This would represent a large number of occasions when an AED was retrieved for no benefit. There was a non-

For peer review only - http://bmjopen.bmj.com/site/about/guidelines.xhtml

intentional delay of 1-4 minutes in activating GoodSAM in London after a call-handler established a potential OHCA, which became apparent and was rectified only after completion of this study. Reducing the time between the emergency call receipt and first-responder activation, and other strategies such as providing information of expected arrival times of the statutory ambulance service, may minimise the chances of a responder's assistance not being required once they reach the scene.

Early CPR is vital, so any intervention to improve AED use must not unduly delay CPR. This is possible where AEDs are easily available or if more than one person responds, with one going direct to the patient and another collecting an AED. This is likely to require more volunteers registered with the platform. AEDs can also be dispatched to the scene using taxi drivers (23) or unmanned aerial vehicles (24,25). It is important to continue to strengthen data capture via the GoodSAM app, to accurately record which interventions first-responders perform during an alert.

Strengths and weaknesses of the study

To our knowledge, this is the first study reporting a theoretically-informed analysis of behaviours affecting AED use in a volunteer first-responder system. We have additionally developed potential interventions for testing that all those who operate similar first-responder systems worldwide can consider. As a public-access AED was retrieved on only one occasion, we lack information about the experience of deciding to retrieve and then use a public-access AED, but this provides justification to develop and test interventions to increase AED use.

For peer review only - http://bmjopen.bmj.com/site/about/guidelines.xhtml

Participants occasionally reported technical problems with the app that did not fit into a TDF domain. This means a small amount of information was not coded using the TDF and was not categorised into the COM-B model. However, we shared these findings with GoodSAM for quality-improvement purposes, as they impact first-responders' opportunities to respond effectively.

Interviews were short, but we focussed on one particular action (decision to retrieve an AED) in a group with the same exposure (a GoodSAM alert). Our response rate was low (30/248), so the sample may represent more confident and motivated first-responders. We introduced targeted selection during our second recruitment window because we were over-sampling those who accepted their latest alert. This will have placed a bigger value on reasons for declining alerts and reduced opportunities to identify important facilitators.

However, our recruitment method left us unable to explore the characteristics of those who did not reply to the invitation e-mail. With a sensitive topic, participants may have been more likely to tell the interviewer what he wanted to hear (26). CMS is a clinician and doctoral research student but did not offer clinical advice or feedback. However, this could affect how participants responded (27), or how he interpreted information imparted at interview (28).

Future research

Delivering many of the proposed interventions requires a sufficient number and density of first-responders. We will investigate with stakeholder groups means of doing this,

For peer review only - http://bmjopen.bmj.com/site/about/guidelines.xhtml

including improved recruitment strategies and if the need for prior CPR certification is an absolute for registration with the app. Not all volunteer first-responder systems require this. Indeed, it varies between different ambulance services using GoodSAM – in New Zealand, prior CPR certification is not required to register. Research priority exercises with GoodSAM, local ambulance services and service users will help us prioritise those interventions that could be tested before an increase in responder numbers, such as highlighting AED location and providing access codes to locked cabinets. These allow existing GoodSAM first-responders to make an informed decision about whether or not to retrieve an AED.

The focus of this study was AED use. However, we identified that enabling any intervention before an ambulance arrived and declining an alert were important barriers to overcome to maximise GoodSAM's impact. Although some of our proposed interventions **(Table 1)** address these issues, overcoming these barriers may merit further research.

Conclusion

A first-responder used a public-access AED on one occasion in this study. An AED was already attached to the patient on another occasion, and three others took their own AEDs to the scene. Despite a capability and motivation to use an AED, interview participants perceived a lack of opportunity to do so. Most believed going to the patient first to assess CPR provision was more beneficial to the patient than diverting to retrieve an AED first. Stakeholders may share this view, but there may still be the

For peer review only - http://bmjopen.bmj.com/site/about/guidelines.xhtml

For peer review only

potential to improve AED use without compromising CPR provision, particularly if there are multiple responders available for each alert.

Word count: 4165

For peer review only

For peer review only - http://bmjopen.bmj.com/site/about/guidelines.xhtml

Data Availability Statement: All data relevant to the study are included in the article or uploaded as supplementary information. Transcripts and audio recordings are stored securely in encrypted computer files and are available to the study team if needed.

Author contributions: CMS, FG and GDP developed the study protocol and design of participant information sheets, consent forms and topic guide. IV and RTF subsequently reviewed and edited. GDP provided guidance about clinical relevance of interview questions, FG about framing questions and interview technique. RTF provided specific guidance about the integration and intended use of the GoodSAM app with London Ambulance Service. CMS recruited, conducted interviews and transcribed. CMS and FG analysed interview data. FG and IV provided expert guidance regarding the application of the BCW and development of interventions. CMS drafted the manuscript for both original submission and resubmission, which all authors reviewed and edited.

Funding statement: This study/project is funded by the National Institute for Health Research (NIHR) Doctoral Research Fellowship (DRF 2017-10-095). The views expressed are those of the author(s) and not necessarily those of the NIHR or the Department of Health and Social Care.

Competing interests. CMS is an NIHR Doctoral Research Fellow and this work was part of his PhD. He also has a volunteer role at the Resuscitation Council UK. GDP is a NIHR Senior Investigator and is supported by research grants from the NIHR, Resuscitation Council UK and the British Heart Foundation.

For peer review only - http://bmjopen.bmj.com/site/about/guidelines.xhtml

Acknowledgements: Mark Wilson, co-founder and medical director of GoodSAM, for access to GoodSAM and information relevant to this article and CMS' PhD, Deepti Bal, operations and projects director at GoodSAM, for assistance in identifying and contacting GoodSAM first-responders; and members of CMS' PhD advisory group – Claire Hawkes, Christopher Hartley-Sharpe, Julian Hague (PPI), John Long (PPI) – who provided guidance and review on the project protocol, topic guides and participant information sheets.

For peer review only

References

1. Hawkes C, Booth S, Ji C, Brace-McDonnell SJ, *et al.* Epidemiology and outcomes from out-of-hospital cardiac arrests in England. *Resuscitation* 2017;**110**:133-40. doi:10.1016/j.resuscitation.2016.10.030
2. Holmberg MJ, Vognsen M, Andersen MS, *et al*. Bystander automated external defibrillator use and clinical outcomes after out-of-hospital cardiac arrest: A systematic review and meta-analysis. *Resuscitation* 2017;**120**:77-87. doi: 10.1016/j.resuscitation.2017.09.003
3. Smith CM, Lim Choi Keung SN, Khan MO, *et al.* Barriers and facilitators to public access defibrillation in out-of-hospital cardiac arrest: a systematic review. *Eur Heart J Qual Care Clin Outcomes* 2017;**3**:264-73. doi: 10.1093/ehjqcco/qcx023
4. Perkins GD, Travers AH, Berg RA, *et al.* Part 3: Adult basic life support and automated external defibrillation: 2015 International Consensus on Cardiopulmonary Resuscitation and Emergency Cardiovascular Care Science with Treatment Recommendations. *Resuscitation* 2015;**95**:e43-69. doi: 10.1016/j.resuscitation.2015.07.041
5. Pijls RWM, Nelemans PJ, Rahel BM, Gorgels APM. A text message alert system for trained volunteers improves out-of-hospital cardiac arrest survival. *Resuscitation* 2016;**105**:182-7. doi: 10.1016/j.resuscitation.2016.06.006
6. Ringh M, Rosenqvist M, Hollenberg J, *et al.* Mobile-Phone Dispatch of Laypersons for CPR in Out-of-Hospital Cardiac Arrest. *N Engl J Med* 2015;**372**:2316-25. doi: 10.1056/NEJMoa1406038
7. Brooks SC, Simmons G, Worthington H, *et al.* The PulsePoint Respond mobile device application to crowdsource basic life support for patients with out-of-hospital cardiac arrest: Challenges for optimal implementation. *Resuscitation* 2016;**98**:20-6. doi: 10.1016/j.resuscitation.2015.09.392
8. Smith CM, Wilson MH, Ghorbangholi A, *et al.* The use of trained volunteers in the response to out-of-hospital cardiac arrest - the GoodSAM experience. *Resuscitation* 2017;**121**:123-6. doi: 10.1016/j.resuscitation.2017.10.020
9. Barry T, Doheny MC, Masterson S, *et al.* Community first responders for out-of-hospital cardiac arrest in adults and children. *Cochrane Database Syst Rev* 2019;**7**:CD012764. doi: 10.1002/14651858.CD012764.pub2
10. Zijlstra JA, Stieglis R, Riedijk F, *et al.* Local lay rescuers with AEDs, alerted by text messages, contribute to early defibrillation in a Dutch out-of-hospital cardiac arrest dispatch system. *Resuscitation* 2014;**85**:1444-9. doi: 10.1016/j.resuscitation.2014.07.020
11. Ringh M, Fredman D, Nordberg P, *et al.* Mobile phone technology identifies and recruits trained citizens to perform CPR on out-of-hospital cardiac arrest victims prior to ambulance arrival. *Resuscitation* 2011;**82**:1514-8. doi: 10.1016/j.resuscitation.2011.07.033
12. National Institutes of Health (June 14th 2019). The Scandinavian AED and Mobile Bystander Activation Trial (SAMBA). Available from: https://clinicaltrials.gov/ct2/show/NCT02992873 [Last accessed 27th January 2020].
13. Atkins L, Francis J, Islam R, *et al.* A guide to using the Theoretical Domains Framework of behaviour change to investigate implementation problems. *Implement Sci* 2017;**12**:77.doi: 10.1186/s13012-017-0605-9
14. Michie S. Making psychological theory useful for implementing evidence based practice: a consensus approach. *Qual Saf Health Care* 2005;**14**:26-33. doi: 10.1136/qshc.2004.011155
15. Cane J, O'Connor D, Michie S. Validation of the theoretical domains framework for use in behaviour change and implementation research. *Implement Sci* 2012;**7**:37. doi: 10.1186/1748-5908-7-37
16. Michie S, Atkins L, West R. The Behaviour Change Wheel: A Guide To Designing Interventions. London: Silverback Publishing, 2014. ISBN: 978-1291846058
17. NHS England (2019). Ambulance Quality Indicators Data 2018-19. Clinical Outcome Spreadsheets. Available from: https://www.england.nhs.uk/statistics/statistical-work-areas/ambulance-quality-indicators/ambulance-quality-indicators-data-2018-19/ [last accessed 27th January 2020].

For peer review only - http://bmjopen.bmj.com/site/about/guidelines.xhtml

18. Francis JJ, Johnston M, Robertson C, *et al.* What is an adequate sample size? Operationalising data saturation for theory-based interview studies. *Psychol Health* 2010;**25**:1229-45. doi: 10.1080/08870440903194015
19. Mason M. Sample Size and Saturation in PhD Studies Using Qualitative Interviews. Forum: Qualitative Social Research. 2010;**11**(3). doi: http://dx.doi.org/10.17169/fqs-11.3.1428
20. Michie S, Richardson M, Johnston M, *et al.* The behavior change technique taxonomy (v1) of 93 hierarchically clustered techniques: building an international consensus for the reporting of behavior change interventions. *Ann Behav Med* 2013;**46**:81-95. doi: 10.1007/s12160-013-9486-6
21. O'Brien BC, Harris IB, Beckman TJ, *et al.* Standards for Reporting Qualitative Research: A Synthesis of Recommendations. *Acad Med* 2014;**89**:1245-51. doi: 10.1097/ACM.0000000000000388
22. Zijlstra JA, Beesems SG, De Haan RJ, Koster RW. Psychological impact on dispatched local lay rescuers performing bystander cardiopulmonary resuscitation. *Resuscitation* 2015;**92**:115-21. doi: 10.1016/j.resuscitation.2015.04.028
23. London Ambulance Service NHS Trust (August 2018). Taxi drivers get life-saving training for ambulance pilot scheme. Available from: https://www.londonambulance.nhs.uk/2018/08/01/taxi-drivers-get-life-saving-training-for-ambulance-pilot-scheme/ [last accessed27th January 2020].
24. Boutilier J, Brooks S, Janmohamed A, *et al.* Optimizing a Drone Network to Deliver Automated External Defibrillators. *Circulation* 2017;**135**:2454-65. doi: 10.1161/CIRCULATIONAHA.116.026318
25. Claesson A, Bäckman A, Ringh M, *et al.* Time to Delivery of an Automated External Defibrillator Using a Drone for Simulated Out-of-Hospital Cardiac Arrests vs Emergency Medical Services. *JAMA* 2017;**317**:2332-4. doi: 10.1001/jama.2017.3957
26. Tourangeau R, Yan T. Sensitive questions in surveys. *Psychol Bull* 2007;**133**:859-83.doi: 10.1037/0033-2909.133.5.859
27. Britten N. *Qualitative Interviews*. In: Pope C, Mays N, editors. Qualitative Research in Health Care. 3rd ed. Oxford: Blackwell Publishing, 2006. p. 12-20. ISBN: 978-1405135122
28. Ormston R, Spencer L, Bernard M, Snape D. *The Foundations of Qualitative Research.* In: Ritchie J, Lewis J, McNaughton Nicholls C, Ormston R, editors. Qualitative Research Practice: A Guide for Social Science Students and Researchers. 2nd ed. London: SAGE, 2014. p. 1-25. ISBN: 978-1446209127

For peer review only - http://bmjopen.bmj.com/site/about/guidelines.xhtml

Table 1: Potential interventions to increase AED use in GoodSAM first-responders

What needs to change (behavioural determinants)	Potential intervention	Intervention function	Policy category	Potential behavioural change techniques	Mode of delivery
Have the physical skills to use an AED effectively **(Capability)**	Deliver digital CPR/AED training	Training	Service provision: provide a specific training resource<br>Regulation: GoodSAM could require completion of training for continued registration with app	Demonstration of behaviour<br>Feedback on behaviour<br>Behavioural practice / rehearsal	Online or in-app training (e.g. interactive video-training package 'Lifesaver' (https://life-saver.org.uk)
Know about the existence of public-access AEDs **(Capability)**	Provide reminders about nearby AED locations	Education	Communication / marketing: provide regular reminders about AED locations<br>Guidelines: introduce recommendations about checking for AED locations into GoodSAM code of conduct	Prompts / cues	Information via e-mail<br>Visual prompt in-app<br>Audio/voice prompt in-app<br>*Delivered at regular, spaced intervals – **not** at the time of an alert*
Be aware of nearby public-access AED locations **(Opportunity)**	Highlight the location of the nearest AED at the time of the alert	Enablement	Environmental / Social planning: designing the in-app environment to enable people to recognise the location of the nearest AED	Prompts / cues	Visual prompt in-app<br>Audio/voice prompt in-app<br>*Delivered during an alert*
Be able to locate and retrieve an AED that is close enough and accessible **(Opportunity)**	Provide access codes to AEDs in locked cabinets	Environmental Restructuring	Guidelines: Recommendations about how to access public-access AEDs<br>Regulation: Voluntary agreement with ambulance services and other AED custodians to provide access codes<br>Legislation: National-level laws to facilitate access to locked cabinets, e.g. mandated provision of access codes	Prompts / cues	Visual prompt in-app<br>Audio/voice prompt in-app<br>*Delivered during an alert, specific to relevant and local AEDs only*

For peer review only - http://bmjopen.bmj.com/site/about/guidelines.xhtml

Be able to locate and retrieve an AED that is close enough and accessible **(Opportunity)**	Provide standardised information to show custodians of public-access AEDs	Enablement	Guidelines: provide a document to GoodSAM first-responders with recommended form of words<br>Communication / marketing: information campaign targeting AED custodians	Information about health consequences<br>Salience of consequences<br>Information about others' approval<br>(Use of a) Credible source	Print media campaign<br>Digital media campaign<br>Printed card (to leave with custodian)<br>Visual display in-app (to show custodian)
Arrive at the patient before the ambulance service **(Opportunity)**	Streamline location and travel route information at the time of an alert	Enablement	Communication / marketing: deliver 'real-time' information to GoodSAM responder via the app<br>Environmental / social planning: designing in-app environment to enable recognition of AED location and incident	Prompts / cues	Visual prompt in-app<br>Audio / voice prompt in-app<br>*Delivered during an alert*
Have access to one's own AED **(Opportunity)**	Equip first-responders with their own AEDs	Enablement	Regulation: rules designating which GoodSAM responders should be equipped with an AED and how they should respond with it<br>Guidelines: how and when to carry and respond with AED	Adding objects to environment<br>Action planning<br>Review behaviour goal<br>Review outcome goal	Information about appropriate use of AED delivered with equipment<br>Supplementary information via e-mail or via the app
Believe that it is appropriate to retrieve an AED **(Motivation)**	Provide reminders about appropriate use of public-access AEDs	Education	Communication / marketing: reminders re: appropriate AED use<br>Guidelines: recommendations about appropriate situations for AED use	Information about health consequences<br>Information about social and environmental consequences<br>Salience of consequences	Information via e-mail<br>Information via app<br>*Delivered at regular, spaced intervals – **not** at the time of an alert*

For peer review only - http://bmjopen.bmj.com/site/about/guidelines.xhtml

For peer review only

Believe that it is appropriate to retrieve an AED **(Motivation)**	Provide information about route and time to the nearest public-access AED(s)	Persuasion	Communication / marketing: deliver 'real-time' information to GoodSAM responder via app<br>Environmental / social planning: designing in-app environment to display AED and incident location	Information about social and environmental consequences	Visual prompt in-app<br>Audio / voice prompt in-app
Believe that it is appropriate to retrieve an AED **(Motivation)**	Send some responders (if multiple responders available) to retrieve a public-access AED first	Persuasion	Communication / marketing: sharing information about each responders' actions (so all involved know that someone is retrieving AED and someone is going to scene)<br>Guidelines: explaining why GoodSAM responder(s) should retrieve AED first when asked<br>Regulation: rules on when GoodSAM responder(s) should retrieve AED first	Information about social and environmental consequences	Website Code of Conduct<br>E-mail updates<br>*Delivered at regular, spaced intervals – **not** at the time of an alert*<br><br>Visual prompt in-app<br>Audio / voice prompt in-app<br>*Delivered during an alert*
Believe that one can retrieve an AED and reach patient before the ambulance service **(Motivation)**	Provide time-to-patient and ambulance response-time estimates at the time of an alert	Persuasion	Communication / marketing: deliver 'real-time' information to GoodSAM responder via app<br>Regulation: organisational rules about how to safely obtain this information from ambulance services and provide it to GoodSAM responders	Information about social and environmental consequences<br>Salience of consequences<br>Credible source	Visual prompt in-app<br>Audio / voice prompt in-app<br>*Delivered during an alert*
Be confident to use an AED effectively **(Motivation)**	Deliver motivational messages regarding AED use	Persuasion	Communication / marketing: deliver motivational information directly to responder at time of alert	[Verbal] persuasion about capability	Visual prompt in-app<br>Audio / voice prompt in-app<br>*Delivered during an alert*

For peer review only - http://bmjopen.bmj.com/site/about/guidelines.xhtml

Overcome anxiety and stress about responding or using an AED **(Motivation)**	Offer voluntary debrief after an alert (to increase AED use and/or future response rate)	Enablement	Service provision: survey GoodSAM responders, identify those at risk of psychological harm, and offer appropriate follow-up for those who need it	Social support (emotional)<br>Review behaviour goals<br>Review outcome goals	In-app survey post-alert<br>Face-to-face / telephone follow-up if needed

For peer review only

For peer review only - http://bmjopen.bmj.com/site/about/guidelines.xhtml

Figure Legends

Figure 1: Possible barriers and facilitators to AED use – capability

Figure 2: Possible barriers and facilitators to AED use – opportunity

Figure 3: Possible barriers and facilitators to AED use – motivation

For peer review only

For peer review only - http://bmjopen.bmj.com/site/about/guidelines.xhtml

Facilitators
Previous training in CPR/AED use
Previous real-life experience in CPR/AED use
Good awareness of Public Access Defibrillation
Using the app to check AED locations before an alert
Most participants felt capable and competent to respond to alerts
Barriers
Being less familiar of AED locations in unfamiliar areas
Varying recall of information given during an actual alert
Not recalling whether or not AED location were displayed at time of the alert
Not even considering AED retrieval at the time of the alert

Figure 1: Possible barriers and facilitators to AED use – capability

For peer review only

Facilitators

Having one's own AED (Some first-responders did and had taken them to the scene)

Previously known AED available nearby

AED already present on scene

Barriers

Public-access AEDs

- Perceived to be too far away from first-responder or patient at time of alert
- Perceived difficulty finding exact location
- Less availability during out-of-hours alerts
- Perceived to be inaccessible in locked cabinets

App-specific issues:

- GoodSAM location inaccurate at time of alert
- Alert siren not always heard
- App slow to display incident location once alert accepted

Healthcare professionals unable to leave patients at work to respond

Non-healthcare professionals feeling unable to leave work or dependents

Arriving after the ambulance service

Figure 2: Possible barriers and facilitators to AED use – opportunity

For peer review only

Facilitators
Belief in the GoodSAM project and its benefits to patients
Debrief and follow-up after an alert
Knowledge that other first-responders were responding
Belief in the importance of AED use to patient survival
Planning ahead: finding AED locations
Confidence in AED use: helped by previous training and experience
Sense of professional duty
Barriers
Concerns about managing scene
Concerns about managing non-cardiac arrest patients
Duty of care concerns
Less confidence in abilities
Reduced motivation because of experience during recent alert(s):
Too far from incident
Belief that ambulance service would arrive before them
Arriving after the ambulance service, even if accepting alert promptly
Being less likely to respond overnight
Prioritising arrival of scene and starting CPR as soon as possible over AED use
Not knowing if someone else was providing CPR at scene
Perceived difficulties in negotiation for AED release from its owner / custodian
Uncertainty about correct strategy: retrieve AED first or go direct to patient?
Difficulty explaining need to use AED to other bystanders
Concerns about acting outside normal sphere of work (healthcare professionals)

Figure 3: Possible barriers and facilitators to AED use – motivation

For peer review only

DEFIBRILLATOR USE

BY VOLUNTEER FIRST-RESPONDERS FOR OUT-OF-HOSPITAL CARDIAC ARREST

BACKGROUND:

GoodSAM

Ambulance services can use **GoodSAM** to alert volunteer first-responders to a nearby cardiac arrest

The app provides directions to the patient, and shows nearby public-access defibrillators

QUESTION

Do **GoodSAM** first-responders use defibrillators when alerted? What are the barriers?

METHODS:

Telephone interviews with **30** **GoodSAM** first-responders shortly after an alert

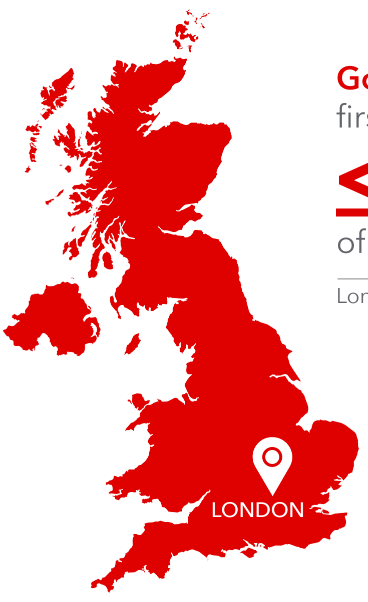

GoodSAM first-responders alerted if **≤300m** of an incident

London, July and November 2018

RESULTS:

14/30 accepted the alert

1 retrieved a public-access defibrillator

3 others took their own defibrillators

We used the **Capability, Opportunity, Motivation Behaviour (COM-B) model** to categorise barriers to defibrillator use

CAPABILITY

Responders were capable of using defibrillators

Didn't think about using them at time of alert

OPPORTUNITY

Defibrillators not close enough to retrieve during an alert

Defibrillators not accessible out-of-hours

MOTIVATION

Responders believed in the benefit of defibrillators

Responders were concerned about delaying CPR by retrieving a defibrillator on the way

We used the **Behaviour Change Wheel (BCW)** to find possible ways of improving defibrillator use, including:

- Highlighting the position of the nearest defibrillator during an alert
- Providing travel routes and times to patient via nearest defibrillator
- Provide access codes to locked defibrillator cabinets
- Equipping volunteers with their own defibrillator

GoodSAM

Higher first-responder density would enable strategies to send some directly to the patient and others to retrieve a defibrillator on the way

For peer review only - http://bmjopen.bmj.com/site/about/guidelines.xhtml

		Reporting Item	Page Number
**Title**			
	#1	Concise description of the nature and topic of the study identifying the study as qualitative or indicating the approach (e.g. ethnography, grounded theory) or data collection methods (e.g. interview, focus group) is recommended	1
**Abstract**			
	#2	Summary of the key elements of the study using the abstract format of the intended publication; typically includes background, purpose, methods, results and conclusions	2-3
**Introduction**			
Problem formulation	#3	Description and signifcance of the problem / phenomenon studied: review of relevant theory and empirical work; problem statement	5-7
Purpose or research question	#4	Purpose of the study and specific objectives or questions	7
**Methods**			
Qualitative approach and research paradigm	#5	Qualitative approach (e.g. ethnography, grounded theory, case study, phenomenolgy,	8-11

For peer review only

		narrative research) and guiding theory if appropriate; identifying the research paradigm (e.g. postpositivist, constructivist / interpretivist) is also recommended; rationale. The rationale should briefly discuss the justification for choosing that theory, approach, method or technique rather than other options available; the assumptions and limitations implicit in those choices and how those choices influence study conclusions and transferability. As appropriate the rationale for several items might be discussed together.	
Researcher characteristics and reflexivity	#6	Researchers' characteristics that may influence the research, including personal attributes, qualifications / experience, relationship with participants, assumptions and / or presuppositions; potential or actual interaction between researchers' characteristics and the research questions, approach, methods, results and / or transferability	**In Discussion (Strengths and Weaknesses)** 22
Context	#7	Setting / site and salient contextual factors; rationale	8
Sampling strategy	#8	How and why research participants, documents, or events were selected; criteria for deciding	9

For peer review only

		when no further sampling was necessary (e.g. sampling saturation); rationale	
Ethical issues pertaining to human subjects	#9	Documentation of approval by an appropriate ethics review board and participant consent, or explanation for lack thereof; other confidentiality and data security issues	12
Data collection methods	#10	Types of data collected; details of data collection procedures including (as appropriate) start and stop dates of data collection and analysis, iterative process, triangulation of sources / methods, and modification of procedures in response to evolving study findings; rationale	9-11
Data collection instruments and technologies	#11	Description of instruments (e.g. interview guides, questionnaires) and devices (e.g. audio recorders) used for data collection; if / how the instruments(s) changed over the course of the study	9-10
Units of study	#12	Number and relevant characteristics of participants, documents, or events included in the study; level of participation (could be reported in results)	13 (reported in results)
Data processing	#13	Methods for processing data prior to and during analysis, including transcription, data entry, data management and security, verification of data	10-11

For peer review only

		integrity, data coding, and anonymisation / deidentification of excerpts	
Data analysis	#14	Process by which inferences, themes, etc. were identified and developed, including the researchers involved in data analysis; usually references a specific paradigm or approach; rationale	11
Techniques to enhance trustworthiness	#15	Techniques to enhance trustworthiness and credibility of data analysis (e.g. member checking, audit trail, triangulation); rationale	10-11
**Results/findings**			
Syntheses and interpretation	#16	Main findings (e.g. interpretations, inferences, and themes); might include development of a theory or model, or integration with prior research or theory	13-21, Figures 1-3, Table 1
Links to empirical data	#17	Evidence (e.g. quotes, field notes, text excerpts, photographs) to substantiate analytic findings	13-21
**Discussion**			
Intergration with prior work, implications, transferability and contribution(s) to the field	#18	Short summary of main findings; explanation of how findings and conclusions connect to, support, elaborate on, or challenge conclusions of earlier scholarship; discussion of scope of application / generalizability; identification of	22, 23-27

For peer review only

		unique contributions(s) to scholarship in a discipline or field	
Limitations	#19	Trustworthiness and limitations of findings	**Box on page 4, 24-25**
**Other**			
Conflicts of interest	#20	Potential sources of influence of perceived influence on study conduct and conclusions; how these were managed	**28**
Funding	#21	Sources of funding and other support; role of funders in data collection, interpretation and reporting	**28**

None The SRQR checklist is distributed with permission of Wolters Kluwer © 2014 by the Association of American Medical Colleges. This checklist can be completed online using https://www.goodreports.org/, a tool made by the EQUATOR Network in collaboration with Penelope.ai

For peer review only
